# High-content screen in human pluripotent cells identifies miRNA-regulated pathways controlling pluripotency and differentiation

**DOI:** 10.1186/s13287-019-1318-6

**Published:** 2019-07-08

**Authors:** Ildercílio Mota de Souza Lima, Josiane Lilian dos Santos Schiavinato, Sarah Blima Paulino Leite, Danuta Sastre, Hudson Lenormando de Oliveira Bezerra, Bruno Sangiorgi, Amanda Cristina Corveloni, Carolina Hassibe Thomé, Vitor Marcel Faça, Dimas Tadeu Covas, Marco Antônio Zago, Mauro Giacca, Miguel Mano, Rodrigo Alexandre Panepucci

**Affiliations:** 1 Laboratory of Functional Biology (LFBio), Center for Cell-Based Therapy (CTC), Regional Blood Center of Ribeirão Preto, Rua Tenente Catão Roxo, 2501, Ribeirão Preto, SP CEP: 14051-140 Brazil; 2 Department of Genetics and Internal Medicine, Ribeirao Preto Medical School, University of São Paulo (FMRP-USP), Ribeirão Preto, SP Brazil; 3 Molecular Medicine Laboratory, International Centre for Genetic and Engineering and Biotechnology (ICGEB), Trieste, Italy; 4 Center for Neuroscience and Cell Biology (CNC), University of Coimbra, Coimbra, Portugal; 5 Department of Biochemistry and Immunology, Ribeirão Preto Medical School, University of São Paulo (FMRP-USP), Ribeirão Preto, Brazil

**Keywords:** Human embryonic stem cells, Pluripotent stem cells, MicroRNA, Cell differentiation, Receptors, Notch, Microscopy, fluorescence

## Abstract

**Background:**

By post-transcriptionally regulating multiple target transcripts, microRNAs (miRNAs or miR) play important biological functions. H1 embryonic stem cells (hESCs) and NTera-2 embryonal carcinoma cells (ECCs) are two of the most widely used human pluripotent model cell lines, sharing several characteristics, including the expression of miRNAs associated to the pluripotent state or with differentiation. However, how each of these miRNAs functionally impacts the biological properties of these cells has not been systematically evaluated.

**Methods:**

We investigated the effects of 31 miRNAs on NTera-2 and H1 hESCs, by transfecting miRNA mimics. Following 3–4 days of culture, cells were stained for the pluripotency marker OCT4 and the G2 cell-cycle marker Cyclin B1, and nuclei and cytoplasm were co-stained with Hoechst and Cell Mask Blue, respectively. By using automated quantitative fluorescence microscopy (i.e., high-content screening (HCS)), we obtained several morphological and marker intensity measurements, in both cell compartments, allowing the generation of a multiparametric miR-induced phenotypic profile describing changes related to proliferation, cell cycle, pluripotency, and differentiation.

**Results:**

Despite the overall similarities between both cell types, some miRNAs elicited cell-specific effects, while some related miRNAs induced contrasting effects in the same cell. By identifying transcripts predicted to be commonly targeted by miRNAs inducing similar effects (profiles grouped by hierarchical clustering), we were able to uncover potentially modulated signaling pathways and biological processes, likely mediating the effects of the microRNAs on the distinct groups identified. Specifically, we show that miR-363 contributes to pluripotency maintenance, at least in part, by targeting NOTCH1 and PSEN1 and inhibiting Notch-induced differentiation, a mechanism that could be implicated in naïve and primed pluripotent states.

**Conclusions:**

We present the first multiparametric high-content microRNA functional screening in human pluripotent cells. Integration of this type of data with similar data obtained from siRNA screenings (using the same HCS assay) could provide a large-scale functional approach to identify and validate microRNA-mediated regulatory mechanisms controlling pluripotency and differentiation.

**Electronic supplementary material:**

The online version of this article (10.1186/s13287-019-1318-6) contains supplementary material, which is available to authorized users.

## Background

A great effort has been devoted to the study of self-renew and pluripotency of embryonic stem cells (ESCs) [1], given their enormous potential in regenerative medicine. A set of core transcription factors (TFs), including OCT4, SOX2, KLF4, and c-MYC (OSKM), sustains pluripotency in ESCs [2, 3] and can reprogram somatic cells into induced pluripotent stem cells (iPSCs) [4, 5].

Distinct from TFs, microRNAs (miRs) are small (~ 22 nt) RNA molecules that act as major post-transcriptional regulators, by binding to partially complementary target mRNAs, blocking their translation and/or leading to their degradation [6]. Despite their small number (2,654 mature miRs, miRBase release 22), they regulate a large fraction of the transcriptome. Their biogenesis involves their transcription as primary miRs (pri-miRs), which assume secondary self-complementary hairpin structures, that are processed in the nucleus by the DROSHA/DGCR8 microprocessor complex giving rise to precursor hairpin miRs (pre-miRs) [7]. These pre-miRs are exported to the cytoplasm and further processed by Dicer to generate the mature miR duplex, composed by one 5′ and 3′ strands (-5p and -3p suffixes in miR names, respectively). This duplex is loaded into the RNA-induced silencing complex (RISC), which contains an Argonaute (AGO) protein, and the passenger strand is removed and degraded (historically referred as star “*” strand), while the guide strand drives the complex to the target mRNAs, inhibiting their translation and/or destabilizing and reducing their transcript levels [6, 8].

Transcriptomic studies have identified miRs specifically expressed in pluripotent cells, and repressed upon differentiation, that could be involved in the maintenance of their undifferentiated properties, as well as miRs with opposite roles, induced upon differentiation [9, 10]. For instance, Stadler et al. found that many miRs members of the miR-302 and miR-371–373 clusters and of the miR-17 family were highly expressed in pluripotent cells and downregulated in most cell lines during differentiation (including hESCs and NT2 cells); conversely, several miRs allocated in specific chromosomal clusters (such as miR-24/miR-27a/miR-23a) were induced during differentiation [10]. Interestingly, pluripotency-related miRs can improve iPSC reprogramming induced by TFs or even completely substitute them [11–15].

Despite the advances in functional genomics, with genome-wide methods able to profile the transcriptome, proteome, and miRNome of pluripotent cells, a true understanding of how these gene products impact cell biology depends on experimental approaches able to systematically identify and quantify molecular and phenotypic events affected by gain or loss-of-function. In this context, high-content screening (HCS) combines the automated acquisition of fluorescence microscopy images of cell-based assays (carried in multiwell plates) with quantitative digital image processing, allowing the simultaneous study of multiple characteristics in stem cells [16]. Specifically, by coupling immunofluorescence of selected markers to nuclear and cytoplasmic fluorescent stains, several morphometric and intensity measurements can be simultaneously quantified at the single-cell level [17, 18], allowing the functional dissection of phenotypic consequences of miR [19]. Up to date, only three small focused screenings have evaluated miR roles in pluripotency, all using a mouse ESC line knockout for DGCR8 [18]. This cell line lacks most endogenous miRs [20–22] and was initially generated to assess the role of DGCR8 in miR processing and to study the global role of miRs in early embryonic development and ES cell differentiation. Although mESCs from Dgcr8^−/−^ embryos are morphologically normal and express ESC-specific markers, they show an extended population doubling time with accumulation in the G1 cell cycle phase and show large differentiation defects, failing to repress pluripotency markers [23]. How results from these screenings translate to cells containing Dgcr8, and human cells, remains to be shown.

With this in mind, our aim was to identify microRNAs and their predicted targeted pathways or processes, contributing to pluripotency or differentiation of human pluripotent cells. To this end, we developed a cell-based assay to investigate, by HCS, the functional effects of 31 miRs differentially expressed between pluripotent and differentiating cells [10, 24], using two of the most studied human pluripotent cell lines in the literature, namely NTera-2 (NT2) embryonal carcinoma (EC) cells, a classical model to study pluripotency [25, 26], and the H1 cell line, the first derived hESCs [1]. These cells share several characteristics, including the expression of pluripotency markers (e.g., OCT4, Nanog) [27–29], miR expression profiles [30], and similar responses upon differentiation induced by all-trans retinoic acid (atRA), including changes in miR expression profile [10], morphology, and expression of pluripotency markers [31]. Importantly, NT2 cells also closely resemble H1 hESCs in terms of cell cycle control [32]. Human and mouse ESCs are characterized by a high proliferation rate and a shortened G1 phase, limiting the differentiation associated with this phase [32–34]. Importantly, the Cdk1-Cyclin B complex seems the only complex with a cell cycle-dependent behavior in mESCs [35] and, in contrast to other cyclins (A, D, or E), Cyclin B1 (CCNB1) is the only cyclin required until implantation of the embryo [36]. Cyclin B levels increase during late-S across G2 and M phases and drastically drop upon re-entry into G1, when it abruptly translocates into the nucleus (shortly before nuclear envelope breakdown), being degraded during G1 [37]. Components of the Cyclin B1 pathway are centrally involved in the dissolution of the pluripotent state, strongly connecting G2 phase with pluripotency [38].

With that in mind, in addition to Oct4 and several morphometrical parameters, we evaluated Cyclin B1 as a surrogate marker of the undifferentiated status associated with the G2 phase of the cell cycle in pluripotent cells. By clustering miRs based on the induced multiparametric phenotypic profiles, and by applying enrichment analysis to the sets of predicted targets shared by clusters of miRs inducing similar phenotypes, we were able to uncover signaling pathways and biological processes potentially mediating their effects on pluripotency and differentiation. Specifically, we show evidence that miR-363-3p decreases the transcript levels of its predicted targets, the receptor NOTCH1 and PSEN1 (a Y-secretase component mediating Notch activation), counteracting the differentiation induced by the Notch ligand DLL1.

## Methods

### Study design (HCS assay and screening)

Detailed supplemental procedures can be found in an additional file (see Additional file 1). Given the lack of systematic studies evaluating the function of miRs in human pluripotent cells, we choose to functionally evaluate a selected set of 31 miRs previously shown to be differentially expressed between undifferentiated and differentiating pluripotent human cells (see Additional file 2: Table S1). These miRs were found to be induced (11 miRs) or repressed in nine hESC lines (including H1 and H9) and NT2 cells, upon transference from a condition favoring their undifferentiated state to a condition promoting a rapid shift toward multilineage differentiation [10]; most miRs were initially cloned and characterized in mESCs [39] and/or hESCs [40]. Additionally, part of the selected pluripotency-related miRs were expressed at higher levels in mESCs and miPSCs (as compared to mouse embryonic fibroblasts-MEFs), had promoters associated with Oct4/Sox2/Nanog/Tcf3, and also were actively transcribed during miPSC reprogramming [24].

To extend the relevance of this screening, we used two classical human pluripotent cell lines, NT2 EC cells [25, 26] and H1 hESCs [1], which share several features, including the expression of pluripotency markers (e.g., OCT4, Nanog) [27–29], and cell cycle characteristics, with a shortened G1 phase [32], associated with the accumulation of cells in G2 phase expressing high levels of cyclin B1 [35, 37], which directly links cell cycle and pluripotency [38].

**Fig. 1:**
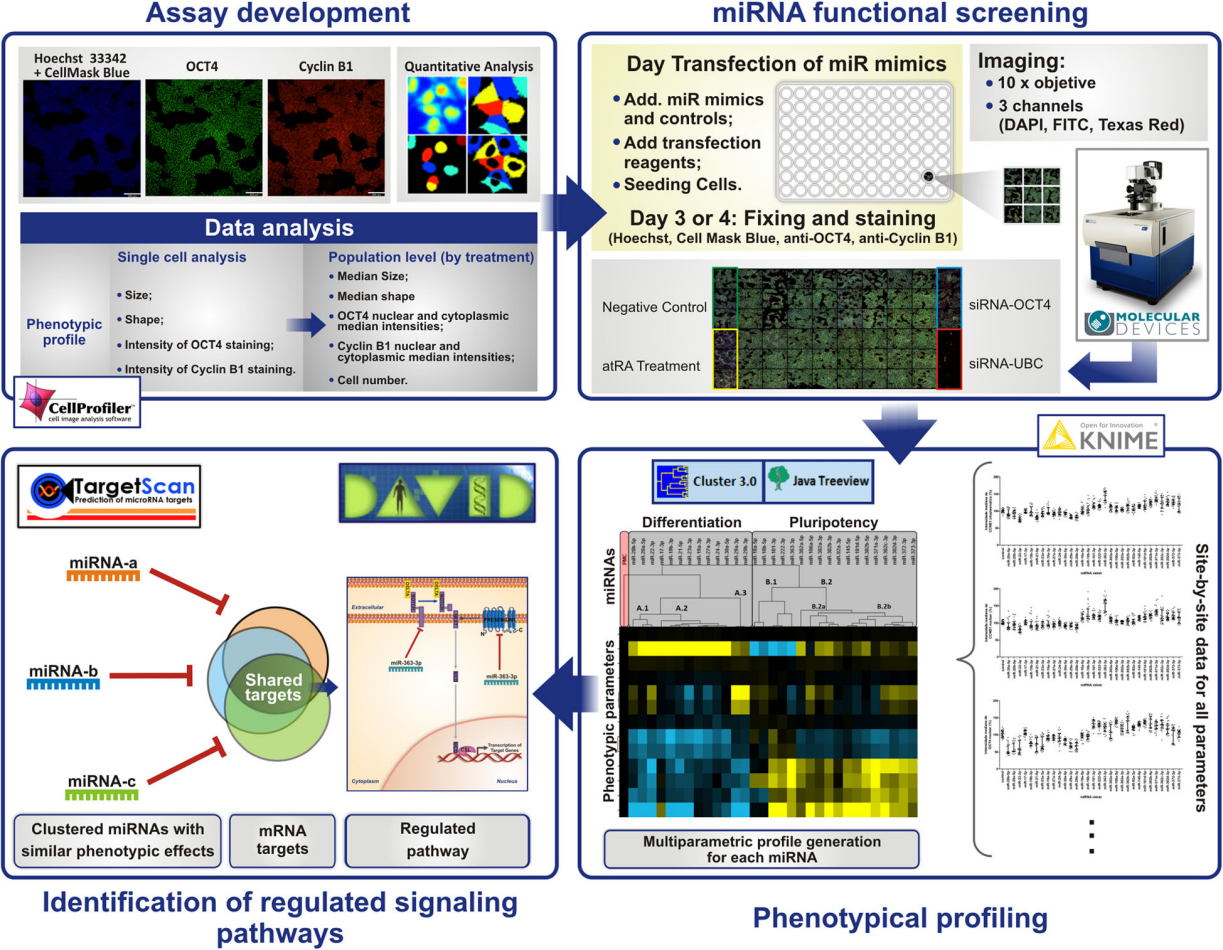
General outline of the study. First: A high-content screening (HCS) assay based on H1 hESCs and NTera-2 cells was developed, to explore pluripotency and differentiation. Several morphometrical features, as well as intensity-related measurements (from OCT4 and Cyclin B1 staining), were obtained from nuclei and cytoplasm compartments. Second: The HCS assay was used in a miR functional screen in 96-well plates to investigate the effects of 31 miRs. Following transfection and culture, images were acquired with an ImageXpress micro XLS HCS system (Molecular Devices). Third: Following image and data analysis (with CellProfiler and KNIME softwares, respectively), the multiparametric phenotypic profile describing the functional effect of each miR was submitted to hierarchical clustering (using Cluster 3.0 and Java Treeview), allowing the identification of miRs with similar functional effects (pro-pluripotency or pro-differentiation). Fourth: TargetScan was used to identify predicted targets shared by miRs in the same cluster (i.e., inducing similar phenotypes). Signaling pathways and biological processes enriched for these shared targets were identified using the DAVID tool

Based on the above, we designed a cell-based assay, compatible with HCS, to simultaneously evaluate morphological and molecular changes related to pluripotency, cell cycle status, and differentiation (Fig. 1, top left panel). Cells were immunostained for OCT4 and Cyclin B1 and counterstained with nuclear (Hoechst) and cytoplasm (HCS CellMask Blue) fluorescent dyes. This allowed the quantitative measurement of several morphometrical as well as intensity features of the markers OCT4 and Cyclin B1 at the single-cell level, in the nuclei and cytoplasm compartments; stainings were optimized so that segmentation of the nucleus and cytoplasm could both be performed in the blue channel, respectively as high and medium fluorescence intensity objects. We used this assay to carry two functional miR screens, using H1 hESCs and NT2 EC cells (Fig. 1, top right panel). For each screening, two 96-well plates were used to accommodate triplicates of the 31 miR mimics evaluated plus negative controls (miR-Ctr, a miR without targets in the human transcriptome) and positive controls for differentiation (atRA and esiRNA-OCT4), as well as a lethal siRNA (siRNA-UBC, targeting the essential gene UbiquitinC) to control for transfection efficiency. Following reverse transfection and culture for 3–4 days, automated image acquisition (9 sites per well, × 10 objective) was performed using an ImageXpress micro XLS HCS system (Molecular Devices) and image and data analysis was performed using CellProfiler and KNIME open-source softwares.

Transfection efficiency was evaluated by comparing the total number of cells (nucleus counts) in wells with cells transfected with a lethal siRNA-UBC control, to those transfected with control miR-Ctr. To further evaluate if both plates of each screening were comparable, for each plate, we evaluated the reduction of nuclear OCT4 staining (as compared to cells transfected with miR-Ctr) in the wells with cells transfected with esiRNA against OCT4 or treated with 10 μM atRA.

Concentration of staining reagents (dyes or antibodies) was defined following titration, in order to guarantee a non-limiting excess of reagents, i.e., the reagent concentration tested, in which there is no quantifiable increase in staining intensity, as compared to the lower concentration tested. Of notice, automation of image acquisition and analysis with the exact same parameters, an inherent characteristic of the HCS approach, guaranties an unbiased and reproducible quantitation, thus allowing the results obtained from distinct wells to be compared [41, 42]. In addition, in order to further validate the quantitative results obtained by the antibodies used in our HCS assay, we compared them to those obtained with distinct antibodies (a total of three anti-OCT4 and two anti-cyclin B1 antibodies). Moreover, western blotting was used to further validate the specificity of selected antibodies and, also, to compare how the quantitative results obtained by HCS relate to those obtained by densitometric analysis of western blot protein bands. A detailed description can be found in the “Antibody validation” section of the supplemental procedures (Additional file 1).

### Image and data analysis

Image and data analysis was carried out using the softwares CellProfiler (cellprofiler.org) [43, 44] and KNIME (knime.com) [45]. Once segmented, several morphometrical features, as well as intensity-related measurements, were obtained from the nucleus and cytoplasm of each cell. Moreover, in order to minimize experimental plate-to-plate variation, we carried a normalization using the miR-Ctr wells (present in both plates) as references. The resulting normalized values are represented as *percentage of control* (POC), allowing a direct comparison of all treatment conditions in both plates of each screening [45]. Median values from each quantified parameter were combined in a multiparametric phenotypic profile representing the effect of each miR in the whole population. Details are provided in the supplemental experimental procedures (see Additional file 1).

### Phenotypic clustering of miRs, identification of shared predicted targets, and pathway analysis

In order to obtain a less redundant and more naturally interpretable set of biologically relevant phenotypic parameters, the following features were selected to compose multiparametric phenotypic profiles: cell count, solidity (a feature varying from 0, for complex shapes with reentrances, up to 1, for solid shapes), eccentricity (varying from 0 to 1, from round to increasingly elliptical shapes), nuclear and cellular areas, nuclear and cellular perimeter, and nuclear and cytoplasmic OCT4 and CCNB1fluorescence intensities. The phenotypic profiles obtained for all miR treatments were submitted to hierarchical clustering using the software Cluster 3.0, using centered correlation metrics and average linkage [46], and heatmaps and clusters were generated and visualized using Java Treeview [47]. Next, we used all predicted mRNA targets of each miR, downloaded from TargetScan Human 7.1 [48], to identify and select transcripts commonly targeted by miRs belonging to the same cluster. Finally, we used the DAVID (V6.8) [49, 50] to identify enriched signaling pathways and biological processes. Details are provided in the supplemental experimental procedures (see Additional file 1).

### Evaluation of miR effects on transcript levels of predicted target

NT2 cells were reverse transfected with selected mimics in 12-well plates and cultured for 2 days prior to RNA extraction. Total RNA was reverse transcribed using High Capacity cDNA Reverse Transcription Kit, and qPCR was performed using either TaqMan Universal PCR Master Mix or Power SYBR Green PCR Master Mix. Relative gene expression was obtained using the 2^-DDCT method [51] (see Additional file 1, detailed supplemental experimental procedures). TaqMan probes and SYBR Green PCR primers used are listed in Table S2 and Table S3, respectively (see Additional file 2).

### Regulation of Notch pathway by microRNAs in pluripotent stem cells

To further dissect the potential repression of Notch signaling by miRs promoting pluripotency features, we obtained a list of genes related to the Notch pathway and identified all miRs targeting them. Four selected miRs with more than ten targets (miR-302c-3p, miR-101-3p, miR-363-3p, and miR-92a-3p) and two miRs with less than five targets (miR-222-3p and miR-371-3p) were individually transfected NT2 cells in 96-well plates and posteriorly co-cultured with OP9-ctrl or OP9-DL1 cells. Cells were fixed and stained for OCT4, nucleus, and cytoplasm and imaged as described before. The nuclear OCT4 intensity was measured using CellProfiler, and the results were compared to the cells transfected with pre-miR negative control and co-cultured with OP9-Ctr. Details are provided in the supplemental experimental procedures (see Additional file 1).

### Statistical analysis

GraphPad Prism 6.0 software (La Jolla, CA, USA) was used for the statistical analysis. All experiments were performed in triplicates. For qPCR data, we used a one-tailed non-parametric Mann-Whitney test. For HCS experiments, we used a non-parametric Kruskal-Wallis test followed with Dunn’s multiple comparison test.

## Results

### Screening quality controls

Transfection efficiency was similarly high in both screening plates for both cell lines (transfection efficiency > 85%), as indicated by the marked reduction in the total number of cells in wells transfected with the lethal siRNA-UBC control, as compared to those transfected with negative miR-Ctr molecules (see Additional file 3: Figures S1 and S2). Additionally, we also evaluated the reduction in OCT4 nuclear intensity following transfection with an esiRNA against OCT4 (as compared to controls wells), allowing us to address the quantitative inter-plate reproducibility of our experimental setting (a sum of transfection efficiency, anti-OCT4 staining, image segmentation, and quantitation of the resulting fluorescence intensity). The reduction in OCT4 nuclear intensity upon transfection with esiRNA-OCT4 (or upon atRA-induced differentiation), as compared to cells transfected with miR-Ctr, was also comparable between both plates in the two screenings (see Additional file 3: Figures S1 and S2). In order to further validate the quantitative results obtained by the antibodies used in our HCS assay, we compared them to those obtained with distinct antibodies using quantitative microscopy. As compared to untreated cells, the relative reduction in the levels of OCT4 and cyclin B1 upon atRA treatment (as quantified by HCS) was strikingly similar for all antibodies used, ranging from 14 to 16%, respectively (see Additional file 3: Figure S3A). Densitometric analysis of western blots carried with proteins extracted from cells cultured in parallel, under the same experimental conditions, was much larger, with a 31–34% reduction in OCT4 levels and 50–53% reduction in cyclin B1 levels (see Additional file 3: Figure S3B). Altogether, these results validate the quantitative results obtained by the antibodies used in our HCS assay and allowed us to confidently proceed with the analysis of the screening results.

### Phenotypic screening in NT2 and H1 cells

**Fig. 2:**
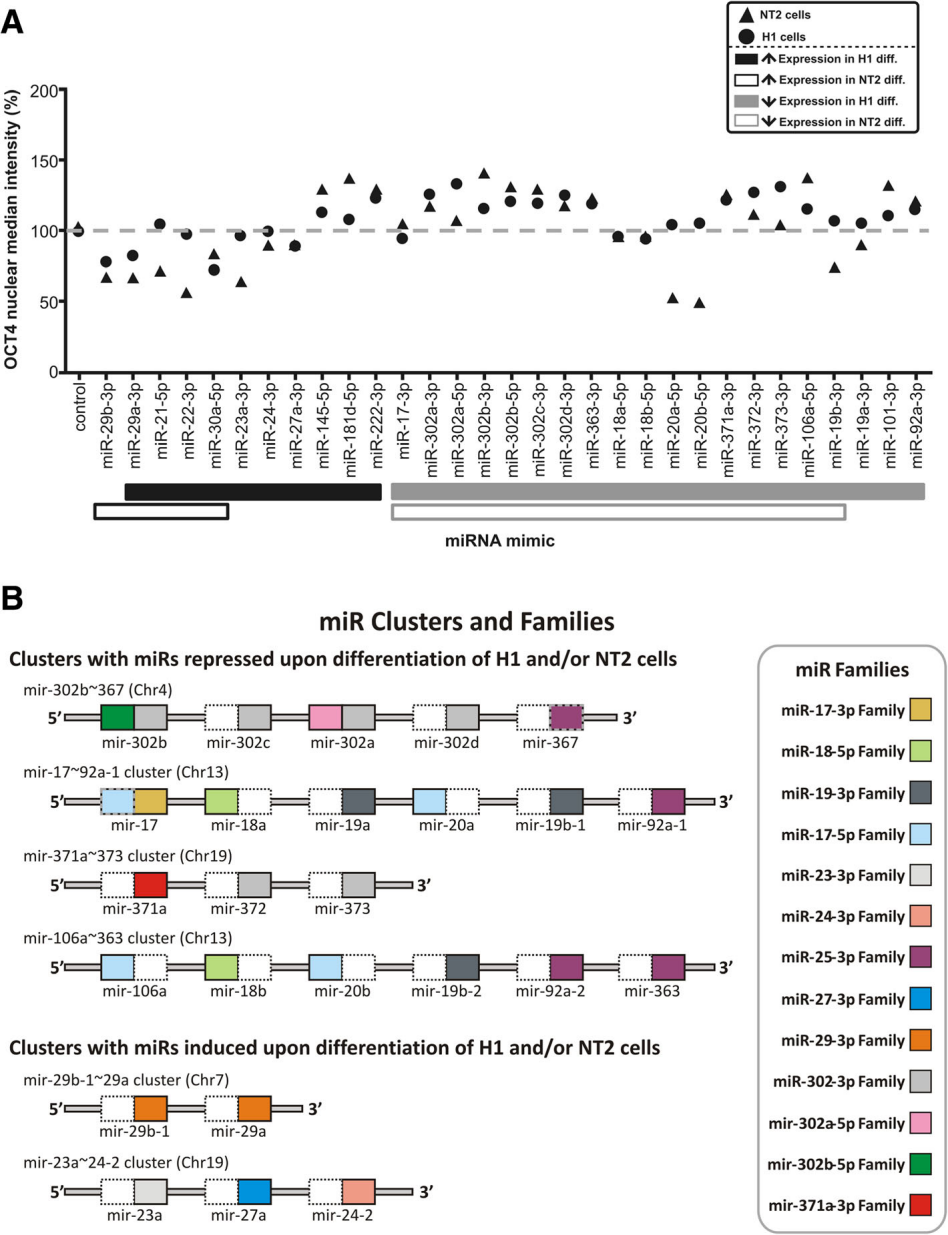
Effects of miRNAs on nuclear OCT4 levels in H1 hESCs and NT2 cells. **a** H1 hESCs and NT2 cells were transfected with miR mimics and the negative control miR-Ctr (PMC) and then submitted to quantitative fluorescence microscopy. OCT4 nuclear median intensity is represented as a percentage relative to the value observed in cells transfected with PMC (NT2 cell depicted as triangles and H1 hESCs as circles). Colored bars in the bottom of the figure indicate the observed change in miRNA expression in H1 and NT2 cells, following induction of differentiation [10]. **b** Graphical representation of selected primary transcripts of miR clusters, with the relative position of the pre-mirs and of the mature miRs. Mature miRs are represented as colored boxes in the 5′ (-5p miR) or 3′ (-3p miR) side of the corresponding pre-mir. Each miR family is represented with a distinct color. Families were defined, as represented in the TargetScan 7 database. For simplicity, only the first representative miR of the family was used in the figure legend. Additional information regarding families can be found in Table S1 (in Additional file 2). Boxes with dotted lines indicate miRs that were not evaluated in our study

For both cell lines, for each miR and each parameter quantified, we obtained 27 values derived from 9 images acquired in each of the 3 replica wells. The median value for OCT4 nuclear median intensity was used to generate Fig. 2a. The primary results quantified for all 27 sites (and all miRs) in NT2 and H1 cells can be seen (respectively) in (Additional file 3: Figure S4A and B), allowing the variability of our HCS assay to be visually accessed. Overall, both cell lines showed similar responses to the evaluated miRs. In general, miRs enriched in pluripotent cells, and whose expression was reportedly downregulated upon differentiation of H1 and/or NT2 cells [10], had a pro-pluripotency effect in our experimental setting, increasing nuclear and cytoplasmic OCT4 and CCNB1 levels. On the other hand, miRs reported as upregulated upon differentiation of H1 and/or NT2 cells [10] had the opposite effect (Fig. 2a). For information on the evaluated miR, regarding their families and the clusters they belong, see (Additional file 2: Table S1) and Fig. 2b.

**Fig. 3:**
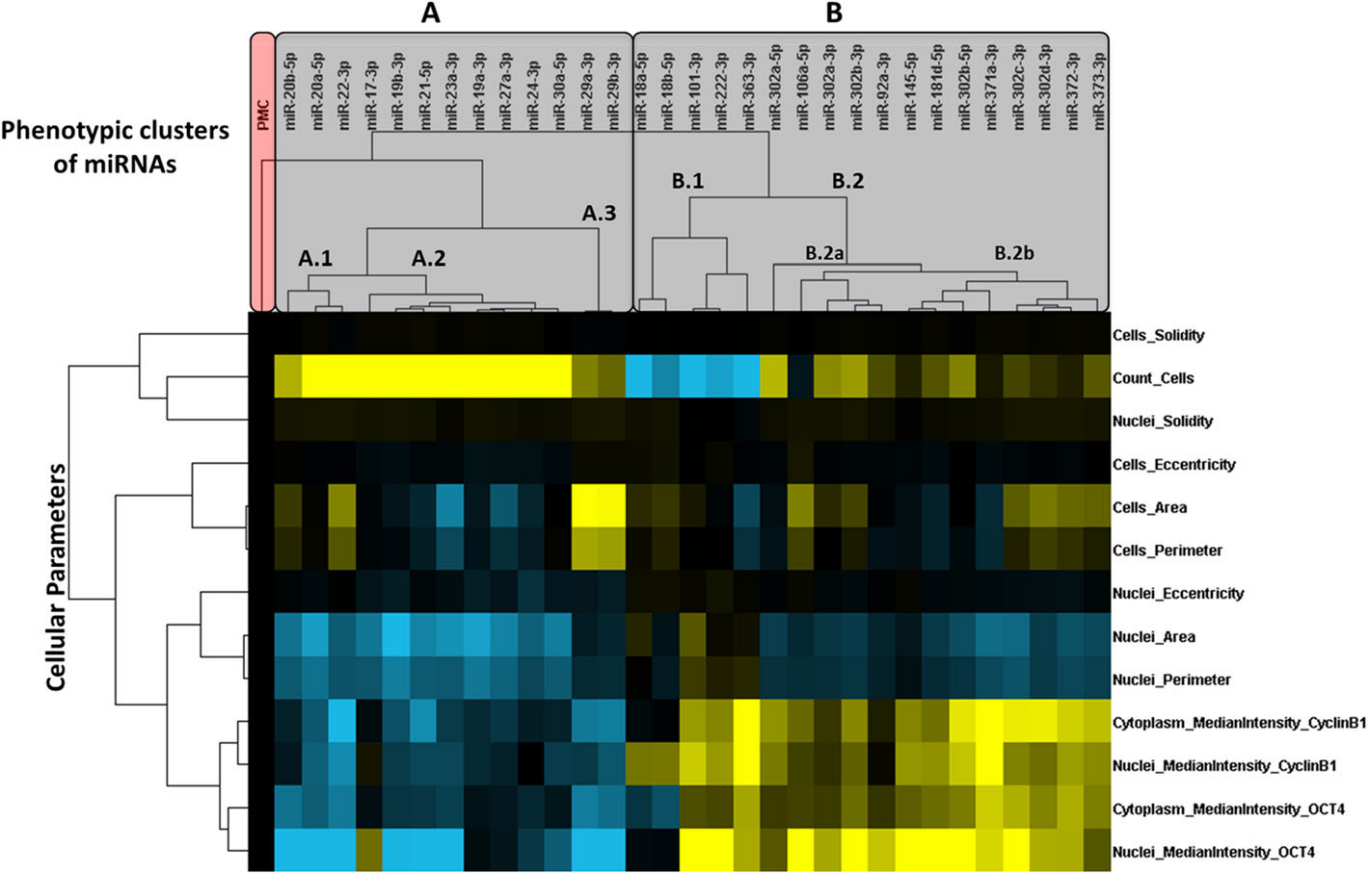
Clustering of miR-induced multiparametric phenotypic profiles in NT2 cells. NT2 cells were transfected with miR mimics and the negative control miR-Ctr (PMC) and then submitted to quantitative fluorescence microscopy. Multiparametric phenotypic profiles specific for each miR were submitted to hierarchical clustering analysis (centered correlation and average linkage). The resulting dendograms above or to the left of the heatmap indicate, respectively, the similarities between the phenotypes induced by miRs (potentially reflecting shared targets and mechanisms of action) or the correlation between distinct phenotypic features. The measurements depicted in the heatmap were calculated as percentage of control (POC) (PMC in black). Highest POC values above or below the control are represented in bright yellow or blue, respectively

**Fig. 4:**
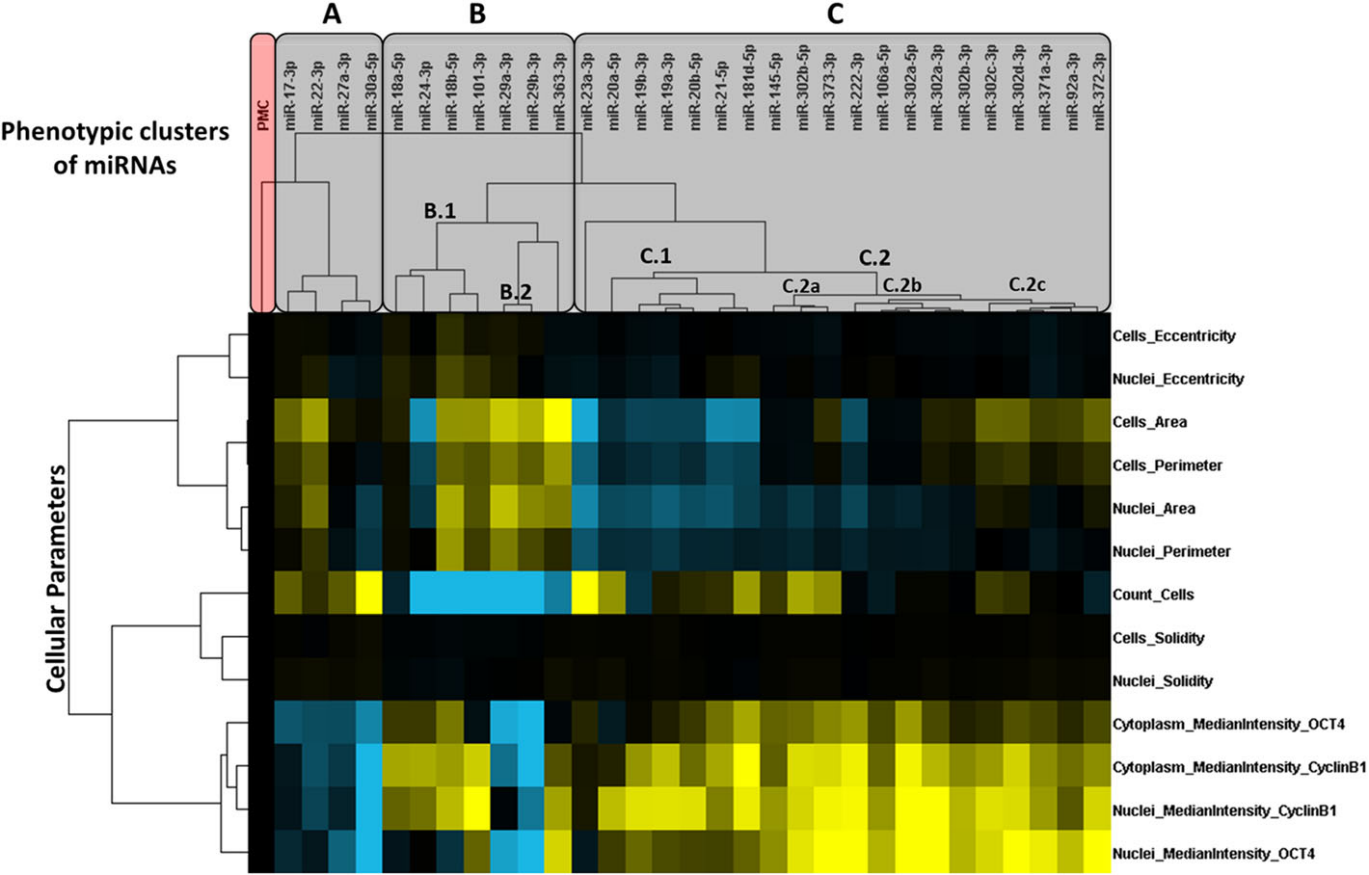
Clustering of miR-induced multiparametric phenotypic profiles in H1 hESCs. H1 hESCs were transfected with miR mimics and the negative control miR-Ctr (PMC) and then submitted to quantitative fluorescence microscopy. Multiparametric phenotypic profiles specific for each miR were submitted to hierarchical clustering analysis (centered correlation and average linkage). The resulting dendograms above or to the left of the heatmap indicate, respectively, the similarities between the phenotypes induced by miRs (potentially reflecting shared targets and mechanisms of action) or the correlation between distinct phenotypic features. The measurements depicted in the heatmap were calculated as percentage of control (POC) (PMC in black). Highest POC values above or below the control are represented in bright yellow or blue, respectively

Following hierarchical clustering of the multiparametric profiles, miRs eliciting similar phenotypic effects were grouped together (for details, see bottom right panel of Fig. 1 and supplemental experimental procedures in Additional file 1). Importantly, median intensity levels of OCT4 and CCNB1 in the nucleus and cytoplasm were found to be highly correlated features, with distinct miRs eliciting similar effects on the levels of both markers (Figs. 3 and 4 and Additional file 3: Figures S4 and S5). This corroborates the assumption that (at least for these microRNAs and cell lines) changes in OCT4 and CCNB1 levels are able to indicate if a given microRNA acts as a pro-pluripotency or pro-differentiation factor.

As can be seen in the clustering results obtained for NT2 cells (Fig. 3) and H1 cells (Fig. 4), the miRs were divided into two major clusters: a cluster containing miRs with pro-differentiation effects (i.e., reducing OCT4 and CCNB1 levels), identified as cluster “A” for both cell lines, and a cluster with pro-pluripotency miRs (i.e., increasing OCT4 and CCNB1 levels), identified as cluster “B” in NT2 cells, and as clusters “B” and “C” in H1 hESCs.

Among miRs enriched in pluripotent cells and downregulated upon differentiation [10], miRs from families miR-17-5p/20-5p/93-5p/106-5p/519-3p/526-3p (specifically, miR-106a-5p from mir-106a~363 cluster) and miR-302-3p/372-3p/373-3p/520-3p (specifically, miR-302a/b/c/d-3p from mir-302b~367 cluster, and miR-372-3p and miR-373-3p from mir-371a~373 cluster), which share very similar seed sequences (AAAGUGC and AAGUGCU, respectively), elicited similar phenotypic effects in both cell lines, markedly increasing nuclear and cytoplasmic OCT4 and CCNB1 levels (clearly indicating a pro-pluripotency effect). Similarly, miR-92-3p and 363-3p, which belong to family miR-25-3p/32-5p/92-3p/363-3p/367-3p (seed AUUGCAC) and originate from the mir-106a~363 cluster (or, also, mir-17~92a-1, in the case of miR-92-3p), also increased OCT4 and CCNB1 levels in both cell lines.

On the other hand, among miRs upregulated upon differentiation of H1 and/or NT2 cells [10], miR-29a/b-3p, from mir-29b-1~29a cluster, induced a marked reduction in OCT4 and CCNB1 levels in both cell lines, as also did miR-30a-5p (Fig. 2).

Importantly, despite large similarities between both cell lines, some differences were clearly observed. For instance, miRs 23a-3p, 27a-3p, and 24-2-3p, from mir-23a~24-2 cluster, only reduced OCT4 and CCNB1 levels in NT2 cells (Figs. 2 and 3), while in H1 this was only evident for miR27a-3p (Figs. 2 and 4). Worth of notice, miRs from this cluster were not induced upon differentiation of NT2 cells (Fig. 2).

One of the most striking differences between the cell lines was the effect elicited by miR-19ab-3p (miR19-3p family) and miR-20ab-5p (also from family miR-17-5p/20-5p/93-5p/106-5p/519-3p/526-3p), deriving from the paralog clusters mir-17~92a-1 and mir-106a~363 (Fig. 2). Despite being repressed upon differentiation of both cell lines, these miRs markedly reduced OCT4 and CCNB1 levels in NT2 EC cells, but had the opposite effect in H1 hESCs. This result specifically highlights how miR-20-5p and miR-106-5p have different effects, despite belonging to the same family. Another unexpected finding was the observed effect of miR-145-5p, miR-181d-5p, and miR-222-3p, which despite being induced upon differentiation of H1 hESCs [10] caused an increase in OCT4 and CCNB1 levels in both cell lines.

Although the two main miR groups, resulting from the hierarchical cluster analysis in both cell lines, successfully separated pro-pluripotency from pro-differentiation miRs, in H1 hESCs, miR-29a/b was grouped with pro-pluripotency miRs in cluster B (instead of cluster A), as a result of its effects on cell morphology and, more importantly, as a result from a marked reduction in cell counts. Interestingly, this group included miR-18a/b-5p, miR-101-3p, and miR-363-3p (all with similar effects in NT2 cells), as well as miR-24-3p. Worth noticing, miR-92a-3p (from the same family as miR-363-3p) did not reduce cell counts in neither of the cell lines. Moreover, while both miRs increased cell size in H1 hESCs, in NT2 cells, they reduced. These findings demonstrate that the cell context largely affects the net phenotypic effect of miRs, even from the same family.

### Identification of pathways targeted by clustered miRs

Given that miRs can have similar phenotypic effects by targeting common components of a given signaling pathway (or biological process) or, alternatively, by targeting distinct components of the same pathway or process [52, 53], we identified the predicted mRNA targets of each miRNA, using TargetScan Human 7.1 [48] and selected transcripts commonly targeted by miRs belonging to the same cluster. Finally, we used the Database for Annotation, Visualization and Integrated Discovery (DAVID) [49, 50] to identify signaling pathways and biological processes enriched for the set of shared targets (see Additional file 4, an Excel file with all results from the pathway analyses), which potentially correspond to the most relevant pathways (Fig. 1, bottom left panel).

**Fig. 5:**
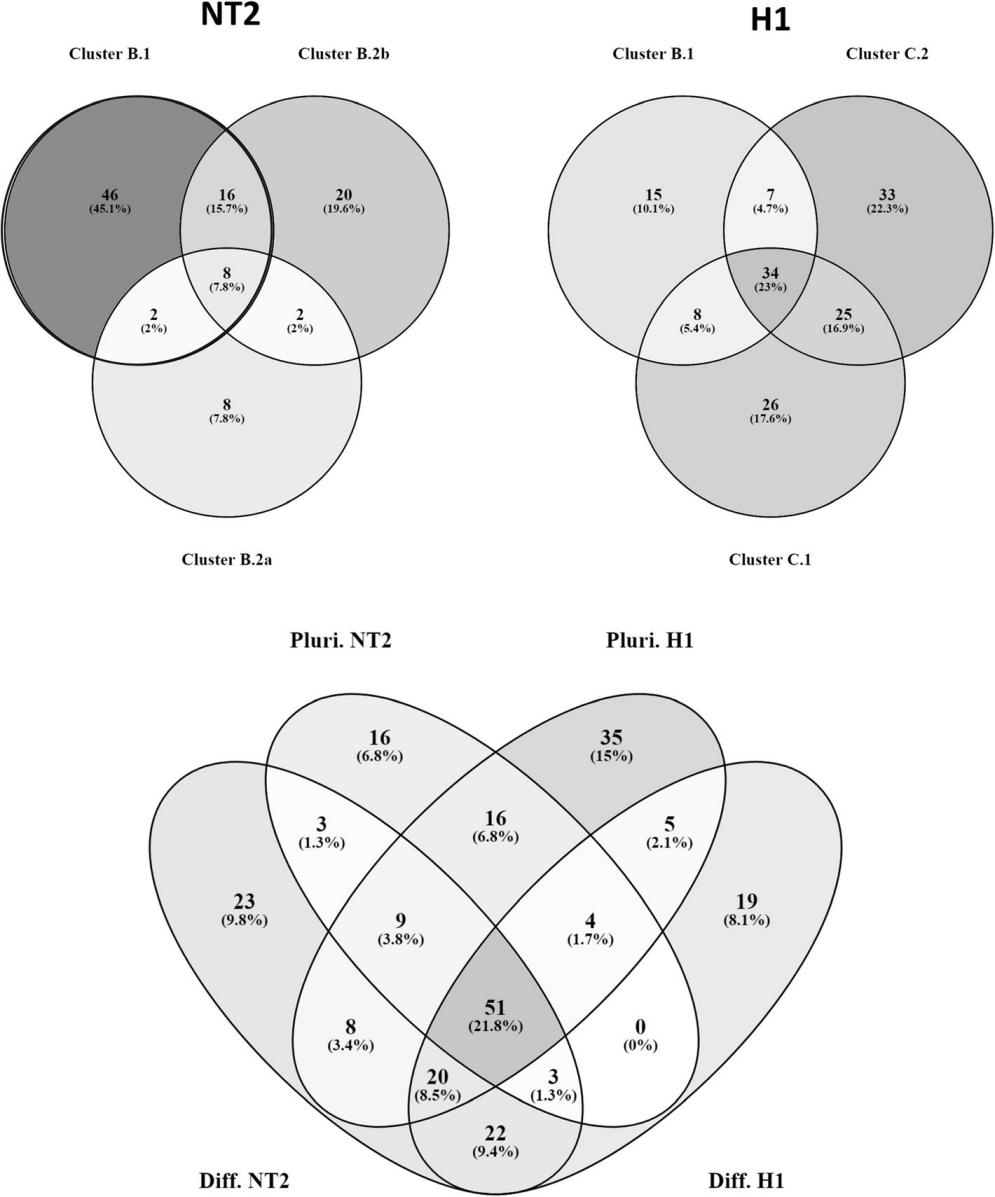
Comparison of identified miR-regulated pathways in NT2 and H1. Predicted targets were identified using TargetScan, and those shared by clustered miRs were submitted to an enrichment analysis using the DAVID tool, allowing the identification of pathways and processes potentially regulated by these miRs at the post-transcriptional level. Top Venn diagrams: comparison of the identified pathways for all pro-pluripotency clusters in NT2 (upper-left) and H1 (upper-right) cells. Bottom Venn diagram: comparison of all the pathways identified for pro-pluripotency (Pluri. NT2 and Pluri. H1) and pro-differentiation clusters (Diff. NT2 and Diff. H1) in both cell lines. Venn diagrams were generated using Venny 3.0

We next compared the pathways identified for each of the three phenotypic clusters with pro-pluripotency characteristics in NT2 cells. From 102 potentially regulated pathways, 45.1% were exclusive for subcluster B.1, 7.8% for subcluster B.2a, and 19.6% for subcluster B.2b (top left Venn diagram in Fig. 5). On the other hand, from 148 pathways identified for the pro-pluripotency clusters in H1 hESCs, 10.1% were exclusive for subcluster B.1, 17.6% for subcluster C.1, and 22.3% for subcluster C.2 (top right Venn diagram in Fig. 5). Interestingly, some pathways were shared by all clusters, like FoxO, mTOR, and DICER pathways. A similar analysis was carried for the pathways identified for the pro-differentiation clusters in both cell lines (see Additional file 5, an Excel file with all pathway comparisons).

Next, we compared all the pathways identified for pro-pluripotency and pro-differentiation clusters in both cell lines (bottom Venn diagram in Fig. 5). Interestingly, among pathways exclusively enriched for targets of pro-pluripotency-related miRs, we found one describing “Presenilin action in Notch and Wnt signaling.” Importantly, this pathway was among the pathways exclusively enriched for miR-targets of B.1 clusters, in NT2 and H1 hESCs, which corresponded to pluripotency-related miRs with the distinguishing feature of restricting proliferation.

### miR effects on predicted target transcript levels

**Fig. 6:**
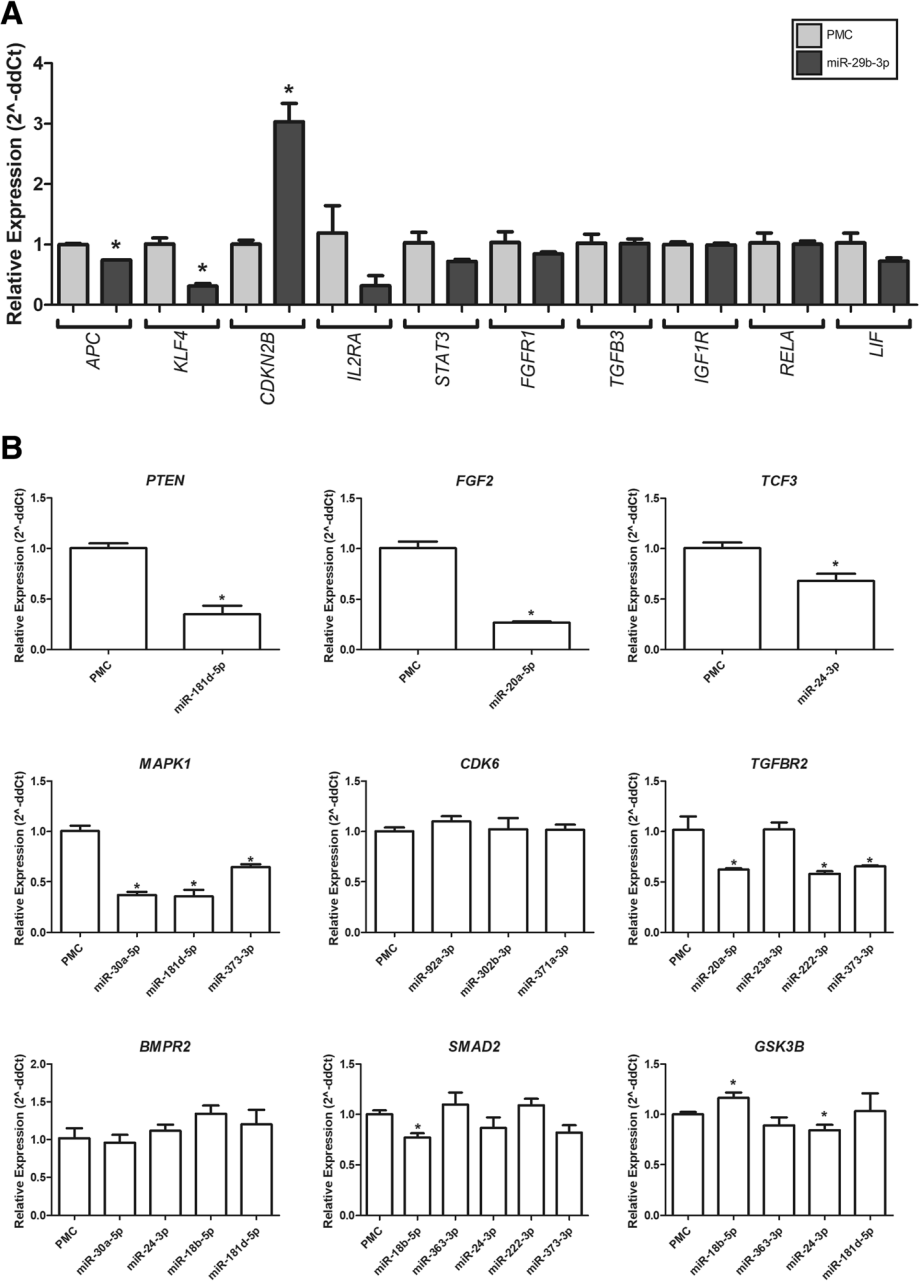
Effects of selected miRs on predicted target levels. NT2 cells were transfected with miR mimics or negative control (PMC), and mRNA levels of predicted targets were evaluated by qPCR after 48 h. **a** Predicted target transcripts for miR-29a/b-3p. **b** Predicted target transcripts from representative miRNAs from identified clusters. Mean expression level of PMC transfected cells were used as reference for the calculation of the relative expression, using the 2^-DDCT method. *p < 0.05 (one-tailed non-parametric Mann-Whitney test)

Given that mammalian microRNAs predominantly act to decrease target mRNA levels [6], in order to evaluate the potential regulation of some predicted targets by selected miRs, we transfected NT2 cells with miR mimics or a control miR and then carried quantitative PCR. From 10 predicted targets of miR-29b-3p (from clusters A.3 and B.2 in NT2 and H1, respectively), a statistically significant decrease (*P* < 0.05), as compared to controls, was only observed for KLF4 and APC. However, a noticeable decrease was also observed for STAT3, IL2RA, FGFR1, and TGFB3 transcripts, whereas CDKN2B were significantly increased, unexpectedly (Fig. 6a). The other representative miRs and corresponding phenotypic clusters in both cells (NT2/H1) were miR-18b-5p (B.1/B.1), miR-20a-5p (A1/C1), miR-23a-3p (A.2/C1), miR-24-3p (A.2/B.1), miR-30a-5p (A.2/A), miR-92a-3p (B.2a/C.2c), miR-181d-5p (B.2b/C.1), miR-222-3p (B.1/C.2b), miR-302a-3p (B.2a/C.2b), miR-363-3p (B.1/B.1), miR-371a-3p (B.2b/C.2c), and miR-373-3p (B.2b/C.2a). It is possible to observe that some miRs caused statistically significant reductions in the levels of their target mRNA transcripts (Fig. 6b). For instance, miR-181d-5p reduced the levels of PTEN and MAPK1, miR-20a-5p of FGF2 and TGFBR2, miR-24-3p of TCF3 and GSK3B, miR-30a-5p of MAPK1, and miR-222-3p and miR-373-3p of TGFBR2.

### miR-363-3p regulates Notch signaling pathway and promotes pluripotency features

The identification of the pathway describing “Presenilin action in Notch and Wnt signaling” as exclusively enriched with predicted targets from pluripotency-related miRs, characteristically restricting the proliferation of NT2 and H1 cells, attracted our attention, and we focused further studies on this pathway.

In order to investigate the potential post-transcriptional regulation of the Notch pathway, we compiled a list of all Notch pathway components using the KEGG database and used TargetScan to find which components were predicted targets of the evaluated miRs (see Additional file 2: Table S4). Next, we selected distinct miRs to functionally test their ability to counteract the differentiation induced by Notch activation. To that end, we transfected NT2 cells with pre-miR negative control (PMC) and co-cultured them with murine OP9-DL1 stromal cells, expressing the Notch activating ligand Delta-Like 1 (DL1) in their surface, or with control OP9 stromal cells (OP9-Ctrl). Additionally, NT2 cells were transfected with selected miRs and co-cultured with OP9-DL1 cells.

**Fig. 7:**
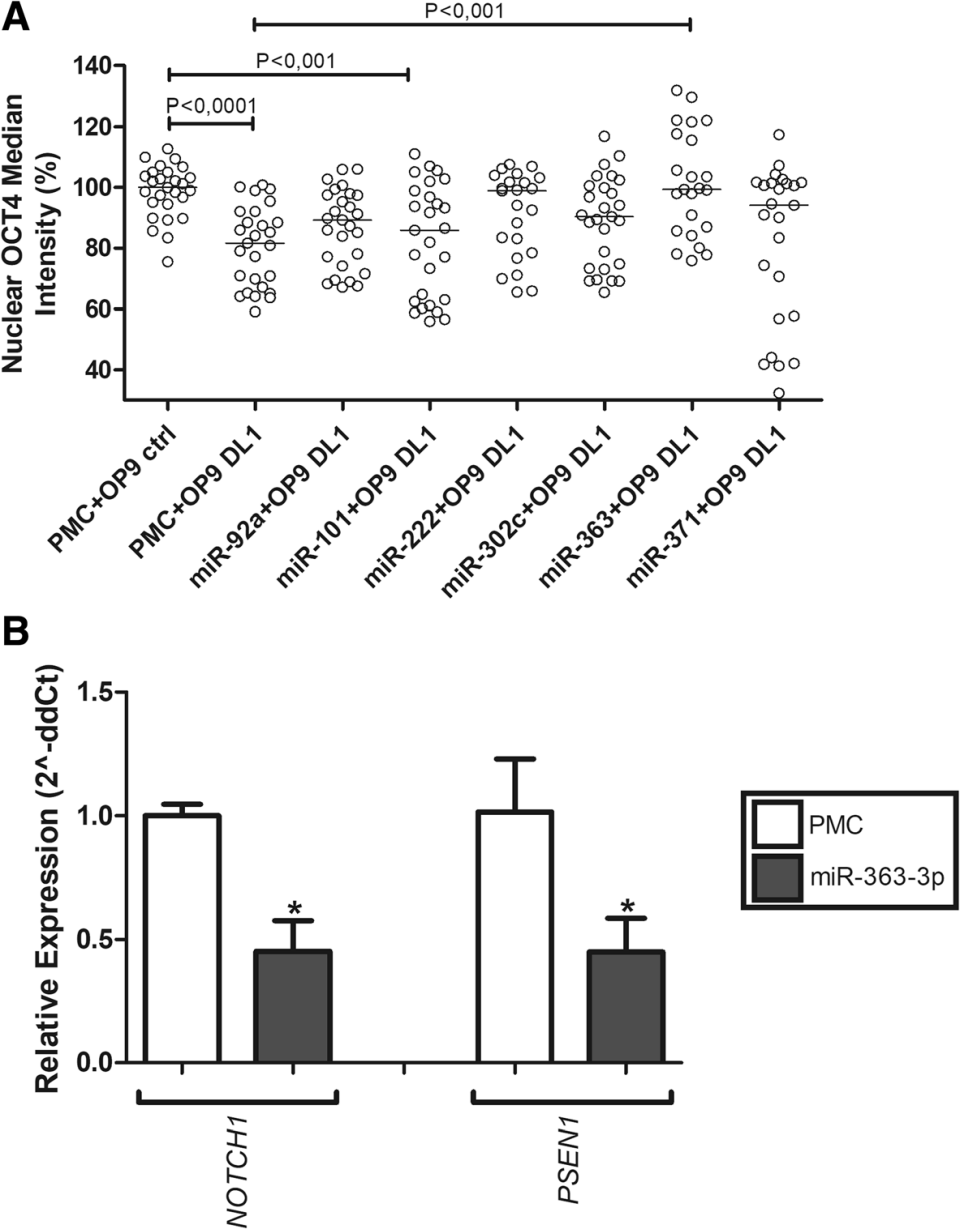
miR-363-3p targets NOTCH1 and PSEN1 and inhibits Notch-induced differentiation in NT2 cells. **a** NT2 cells were transfected with miR mimic and negative control (PMC), and 72 h later, they were co-cultured with OP9-Ctrl or OP9-DL1 stromal cells for more 24 h. Nuclear OCT4 median intensity was then quantified by automated quantitative fluorescence microscopy. Kruskal-Wallis test followed with Dunn’s multiple comparison test. **b** NT2 cells were transfected with miR-363-3p mimics and PMC and *NOTCH1*, and *PSEN1* transcript levels were evaluated 48 h later by qPCR. Mean expression level of PMC transfected cells were used as reference for the calculation of the relative expression, using the 2^-DDCT method. *p < 0.05 (one-tailed non-parametric Mann-Whitney test)

In addition to miR-101-3p and miR-363-3p (pluripotency-related miRs from the B.1 cluster characteristically restricting proliferation in NT2 and H1 hESCs), we selected additional miRs to be used as references, specifically four miRNAs with more than ten targets in the Notch pathway (miR-302c-3p, miR-101-3p, miR-363-3p, and miR-92a-3p) and two miRs with less than five targets (miR-222-3p and miR-371-3p). A statistically significant reduction in OCT4 nuclear median intensity (approximately 20%) was observed in NT2 cells co-cultured with OP9-DL1 cells, as compared to those co-cultured with OP9-Ctrl cells, indicating that Notch signaling induced differentiation (Fig. 7a). Additionally, when comparing the effects of the evaluated miRs in the context of Notch signaling, only miR-363-3p was able to counteract Notch-induced differentiation (i.e., sustain nuclear OCT4 levels comparable to cells co-cultured with OP9-Ctrl cells), as indicated by statistically significant higher nuclear Oct4 levels, as compared to cells transfected with PMC (Fig. 7a). Of notice, of the tested miRs, only miR-92a-3p, miR-101-3p, and miR-363-3p targeted both *NOTCH1* and *PSEN1*, whereas miR-222-3p and miR-302c-3p targeted only PSEN1. Of these, we found that miR-363-3p caused a reduction in *NOTCH1* and *PSEN1* transcript levels, suggesting a miR-induced degradation (Fig. 7b). Given these data, we suggest that miR-363-3p induces pluripotency-related characteristics by repressing Notch pathway activation via degradation of NOTCH1 and PSEN1 mRNA transcripts.

## Discussion

We present the first focused miR functional high-content screen carried in human pluripotent cells. By identifying targets shared by miRs causing similar multiparametric phenotypic profiles, we were able to pinpoint post-transcriptionally regulated signaling pathways involved in pluripotency and differentiation. In particular, we show that the pluripotency-related microRNA miR-363-3p targets *NOTCH1* and *PSEN1* inhibit differentiation mediated by Notch signaling. Moreover, by comparing NT2 cells to H1 hESCs, we show how extremely contrasting effects can result from closely related miRs in the same cell or from the same miR in different cells.

### Similarities and differences between hESCs and NTera-2 cells

Despite overall similarities, some miRs elicited contrasting effects depending on the cellular context. For example, OCT4 and CCNB1 levels were strongly repressed by miR-19a/b-3p and miR-20a/b-5p in NT2 cells, but were robustly induced in H1 cells. In turn, miR-92a-3p and miR-363-3p had opposing effects on cell size, decreasing it in NT2 cells, while increasing it in H1 hESCs. Conversely, some closely related miR (members of the same family) had opposing effects in the same cell line, despite identical seeds. For instance, miR-20a/b-5p and miR-106a-5p showed pro-differentiation and pro-pluripotency effects in NT2 cells, respectively. In turn, miR-92-3p and miR-363-3p had opposite effects on proliferation and nucleus size in the same cellular context, in both cell lines.

### Functional microRNA screening based on DGCR8 KO mESCs shows contrasting phenotypic effect to human pluripotent cells

In order to identify functional differences between the tested miRs in human and mouse cells, we compared our results with those observed in previously published screenings using DGCR8 knockout mESCs [20–22]. The first mESC screening consisted of a MTT assay to specifically search for miRs able to recover the proliferation defect of these cells [20]. From 266 mouse miRs screened, Wang et al. identified members of the miR-290 family (including miR-291a-3p, miR-294, and miR-295) promoting G1 to S phase transition, naming them ES cell–specific cell cycle–regulating (ESCC) miRs. All ESCC miRs belong to the mir-290–295 cluster, homolog to the cluster miR-371-373 in human [54]. Moreover, miRs in this cluster contain seed sequences similar or identical to miRs in the mir-302 and mir-17-92 clusters (AAAGUGC for family miR-17-5p/20-5p/93-5p/106-5p/519-3p/526-3p and AAGUGCU for family miR-302-3p/372-3p/373-3p/520-3p), which also predominate in hESC [55].

Importantly, we found that miR-106a-5p, miR-302a/b/c/d-3p, miR-372-3p, and miR-373-3p had the same effect in H1 hESCs and NT2 cells: increasing proliferation (i.e., cell counts), cell size (i.e., area and perimeter), and expression of OCT4 and CCNB1 in nucleus and cytoplasm (i.e., median intensity) and reducing nucleus size. Of notice, increases in cell size and reduction in nucleus size were unanticipated effects captured only as a result of our multiparametric high-content screen.

In mice, the miR-290~295 cluster is broadly expressed throughout the embryo, but diminishes a few days following implantation, remaining highly expressed in extraembryonic tissues, in line with its roles in implantation and placenta development [56, 57]. In contrast, miR-302a-d cluster is expressed in the embryo proper only after implantation [58, 59]. While deletion of miR-302a-d particularly affects neural development, deletion of miR-290 and miR-302a-d clusters results in early embryonic lethality, revealing redundant functions during early development [60].

Also using DGCR8 KO mESCs, Ma et al. evaluated the effects of 40 microRNAs expressed at higher levels in MEFs and EBs (as compared to mESC) and predicted to target pluripotency-associated transcription factors, including Oct4, Sox2, Nanog, Klf4, c-Myc, Lin28a, Sall4, Rex1, and Stella [21]. Many miRs evaluated in our study were also evaluated by Ma et al., in terms of their effects on Oct4 staining, though not in a quantitative way. Of all 40 miRs, 20 decreased Oct4 staining (including miR-24-3p, 27a-3p, 92a-3p, and 29a-3p), 12 mildly decreased Oct4 levels (including miR-23a-3p), and 8 miRs increased Oct4 intensity (including miR-18a-5p and 30a-5p). Of notice, we observed similar effects on Oct4 nuclear median intensity (in both human cell lines) only for miR-23a-3p, miR-24-3p, 27a-3p, and miR-29a-3p, which decreased Oct4 levels in our screenings. Opposite results were found for miR-92a-3p, which increased Oct4 levels in our screening, and for miR-18a-5p and miR-30a-5p, which repressed Oct4 levels. Nevertheless, our results regarding miR-92a-3p and miR-30a-5p were in line with their expected roles based on the expression behavior in differentiating hESCs [10].

On the other hand, when evaluating mESC colony-forming ability, 17 miRs decreased it (including miR-24-3p, miR-27a-3p, miR-29b-3p), 15 did not affect it (including miR-29a-3p, miR-27b-3p, miR-92a-3p, miR-145a-5p, miR-18a-5p, and miR-30a-5p), and 8 enhanced it (including miR-23a-3p and 23b-3p). Since colony-forming ability derives, in part, from the proliferation and survival of plated cells, we compared it to the miR effects on cell counts obtained by us. In H1 hESCs, we found that miR-24-3p and miR-29a/b-3p decreased cell counts (in line with lower colony numbers), while miR-27a-3p increased cell counts (in contrast to decreased colony numbers). In line with higher colony numbers, miR-23a-3p also increased cell counts in H1 hESCs and NT2 cells. In contrast to the decreased colony formation ability observed by Ma et al., we found that miR-24-3p, miR-27a-3p, and miR-29b-3p increased cell counts in NT2 cells. These contrasts between DGCR8 KO mESCs and H1 hESCs may derive from differences related to the absence of endogenous miRs, but also from differences regarding the pluripotency state of mouse and human ESCs (naïve vs primed, respectively). Importantly, according to Ma et al., miR-27a and miR-24 would exert their pro-differentiation effects, in part, by directly targeting the pluripotency-associated factors Oct4 and Foxo1 and the signal transducers gp130 and Smads [21]. Given our findings, it is likely that the above mechanism may not completely apply to hESCs.

As mentioned before, many miRs able to downregulate AP activity on Dgcr8 knockout mESCs cannot do so on wild-type cells [61]. Additionally, DGCR8 also acts in the maturation of snoRNAs and in the degradation of telomerase RNA [62]. Finally, in the absence of endogenous miRs, the RNAi machinery of DGCR8 KO cells becomes completely available to the exogenously introduced miR, likely resulting in an enhanced effect (including off-target effects of siRNAs). Altogether, our results further stress important differences that compromise a straightforward comparison and translation of the findings obtained with DGCR8 KO mESCs to those obtained with wild-type ESCs, further highlighting the importance of our study. For further discussions, the reader can refer to our review comparing all arrayed genetic screens (siRNAs and microRNAs) carried in the context of pluripotency, reprogramming, and differentiation, published up to date [18].

### microRNAs in naïve and primed pluripotency

In a more recent work, Gu et al. used DGCR8 KO mESCs to evaluate the transition from mESCs in a naive state of pluripotency to epiblast-like cells (EpiLCs) in a primed state. The authors identified 50 upregulated miRs, including those of the miR-302 cluster, while other miRs with similar seeds, such as miR-290 cluster and miR-17-20-106 families, were not altered. By using qRT-PCR, Gu et al. found that miRs from the miR-302 cluster strikingly repressed the expression of naive markers (Rex1 and Klf2) and induced the post-implantation epiblast marker Fgf5; nevertheless, those from the miR-290 cluster also facilitated the exit from naive pluripotency [22]. The role of miR-302 cluster miRs in the transition to the primed state would be in line with its expression following implantation [58, 59]. Moreover, two studies identified microRNAs expressed at higher levels in naive cells of mice or humans (including those from the miR-371-373 cluster in human, or miR-290~295 in mice), as well as those expressed at higher levels in primed cells (miR-302 family and miR-363) [63, 64].

Naïve mESCs, derived from the inner cell mass (ICM) from pre-implantation blastocyst [65], and primed EpiSCs, derived from the implanted blastula epiblast [66, 67], differ in many characteristics. Human ESCs, which are derived from blastocysts from in vitro fertilization, are considered to be more similar to mouse EpiSCs [68]. All miRs from the miR-302-3p family evaluated by us (miR-302a/b/c/d-3p, miR-372-3p, and miR-373-3p) showed conserved functional roles in NT2 and H1 hESCs, increasing proliferation, expression of OCT4/CCNB1, and cell size (and also reducing nucleus size).

Based on these results and given that the miRs enriched in both pluripotency states share similar seeds, it is evident that miRs from the same or related families (but belonging to clusters transcriptionally regulated in an independent manner during development) can have pluripotency-related roles in both naive and primed cells. Moreover, they may likely contribute bi-directionally during naive-primed interconversion, depending on the experimental setting, as exemplified by miRs from the miR-302 and miR-290 clusters facilitating the exit from naive pluripotency [22]. However, given that DGCR8 KO mESC fails to completely silence the naive program and to establish the primed one, it remains to be shown if these findings translate to wild mESCs or hESCs.

### Context-dependent effects of microRNAs on pluripotency and reprogramming

While the pro-pluripotency roles of miR-302 and miR-294 were expected, since they are enriched in pluripotent cells and are repressed during differentiation, the pro-pluripotency role of miR-181 family members is more intriguing. While Stadler et al. [10] found these miRs to be induced during differentiation of hESCs (miR-181b and d) and NT2 cells (miR-181a and b), we found that miR-181d-5p considerably increased OCT4 nuclear levels and cell counts, also reducing cell and nucleus size, in both our screenings. Importantly, ESCC miR mimics from miR-302 and miR-290 clusters and from the miR-181 family all promote the initiation phase during OSK-mediated reprogramming of MEFs into iPSCs [69–71], in line with their pro-pluripotency roles. In mESCs, miR-181 family members are transcribed from three distinct clusters, differentially bound by Oct4, Sox2, Nanog, and TCF3 TFs, and expressed in pluripotent and differentiated cells. While miR-181a-1~181b-2 cluster is bound by all these TFs and expressed in neural precursors and MEFs, miR-181c~181d cluster is bound by all TFs except TCF3 and is expressed only in mESCs; finally, cluster miR-181a-2~181b-1 (in chr1) is expressed only in neural precursors and is not bound by any of these TFs [24]. Interestingly, the differential regulation of these clusters by TCF3 during reprogramming could be implicated in the stage-specific regulation of reprogramming by Tcf3, promoting it in the early phase, while inhibiting in the later stages [72].

MiRs from the miR-181 family are also induced during mESC differentiation, targeting Cbx7, the primary Polycomb ortholog of Polycomb repressive complexes 1 (PRC1) which reads repressive H3K27me3 histone marks left by PRC2, leading to derepression of bivalent genes encoding lineage-specific TFs and markers [2, 73–75]. In contrast, ectopic expression of Cbx7 in mESC enhances self-renewal and inhibits differentiation and X chromosome inactivation; in turn, upon its knockdown, mESC colonies display a flattened morphology [73], characteristics related to the naïve or primed states, respectively. Thus, similar to the miR-302 family, although miR-181 family possesses pluripotency-related properties, it may be associated with the transition to the primed state, by targeting Cbx7.

In addition to miR-181d-5p, miR-145-5p and miR-222-3p were also induced in pluripotent cells upon differentiation [10] and similarly found to considerably enhance OCT4 nuclear levels in both our screenings (increasing cell counts and reducing cell and nucleus sizes). For miR-145-5p, our results were particularly unexpected, as this miR was shown to target OCT4, SOX2, and KLF4, negatively regulating their translation during cell differentiation [76]. Moreover, the inhibition of this miR during reprogramming was shown to increase the efficiency of iPSC generation [77]. These conflicting findings may result from differences between the transient effect mediated by transfection of mimics, as compared to the sustained and strong expression mediated by lentiviral vectors. In fact, contrasting findings arising from different methods used were previously pointed out by our group in the study of the miR-29 family. Using transfection of synthetic miRs or inhibitors, our group and others showed that miR-29a hampers reprogramming by different mechanisms, including activation of WNT/beta-catenin signaling (by targeting GSK3B) and targeting of active DNA demethylation enzymes of the TET family [71, 78, 79]. Strikingly in contrast, expression of miR-29 throughout the process using a retroviral vector promotes reprogramming, an effect resulting from passive global demethylation following constitutive knockdown of the targets Dnmt3a and Dnmt3b [80]. However, transient reduction of DNMT3a/b by siRNAs does not affect reprogramming [81].

Many effects of miRs (such as ESCC miRs) on pluripotency and reprogramming have been attributed to their function in the regulation of key cell cycle regulators [20, 61, 82–84]. In fact, while induction of cell proliferation increases reprogramming efficiency, cell-cycle arrest inhibits it [85]. Thus, many miRs physiologically associated with differentiation of ESCs may promote reprogramming and pluripotency in vitro, trough cell cycle-mediated effects.

### miR-363-3p sustains pluripotency by repressing Notch-induced differentiation

By comparing all the pathways enriched for predicted targets of the pro-pluripotency clusters to those of the pro-differentiation clusters, we noticed that a pathway describing “Presenilin action in Notch and Wnt signaling” was exclusively associated with pluripotency-related clusters, more specifically with those that characteristically restricted proliferation in NT2 and H1 hESCs. This prompted us to explore the potential role of Notch signaling in pluripotency and more specifically to evaluate the potential role of selected miRs in the inhibition of Notch signaling.

In general, Notch signaling begins with the interaction of surface-bound ligands (such as Delta or Jagged) with transmembrane Notch receptors (NOTCH1 to 4), leading to its cleavage (by a gamma-secretase complex containing presenilin, PSEN1) and release of the Notch intracellular domain (NICD), which translocates to the nucleus and forms a complex with the protein RBP-Jk (CSL). This complex binds to DNA *cis*-regulatory elements along with coactivators proteins (including p300 and proteins of the MAML family), activating the transcription of its target genes [86]. As shown by us, Notch signaling (mediated by OP9-DL1 cells) induced differentiation of co-cultured NT2 cells. Moreover, miR-363-3p was able to counteract Notch-induced differentiation, also reducing the transcript levels of its predicted targets PSEN1 and NOTCH1, central players in Notch signaling, in line with the previously reported direct targeting of NOTCH1 by miR-363-3p [87, 88].

Components of the NOTCH pathway are expressed both in hESCs and in NTERA2 cells [89]. Although initial studies concluded that Notch would positively regulate the proliferation of hESCs [90, 91], later studies revealed that this conclusion was likely derived from the unspecific cytotoxic effect of the Notch inhibitor used in these studies, including DAPT [91] and L685458 [90] and, also, by the unspecific activation of Notch by Tripsin/EDTA passaging during culture [92]. In fact, although the Notch pathway is inducible in hESCs, it remains inactive and is not necessary for the propagation of undifferentiated cells, but instead is required for the maintenance of differentiating cells that accumulate in culture [92]. The pro-differentiation role of Notch in hESCs was corroborated in a study showing that Notch signaling inhibition by GSI-18 maintained cells undifferentiated, preventing spontaneous differentiation to all three germ layers, while allowing trophoblastic differentiation [93]. Interestingly, Oct4 physically interacts with RBP-Jk in mESC [94], and siRNA knockdown of OCT4 in H1 hESCs induces the transcription of Notch pathway components and targets, including NOTCH2, TLE2, DTX4, and HES1 [95], suggesting a connection between Notch and Oct4-regulated gene expression.

Interestingly, Annab et al. identified two coexisting subpopulations in hESC, one composed of rounder cells expressing higher levels of Nanog and KLF4 and a more heterogeneous population expressing high levels of Notch components, including NOTCH1, NOTCH2, JAG1, DLL1, HES1, and DTX1 [96]. Given that hESCs chemically reverted to the naive pluripotency state express higher levels of Nanog and KLF4 [97], this could indicate that the expression of Notch components could be associated with a primed population of hESCs. Strikingly, a recent study in search of surface markers capable to distinguish naive and primed states of hESC identified NOTCH receptors 1 and 2 as being specifically expressed in the primed state, while the LIF coreceptor (GP130/IL6ST) was detected exclusively in naive-state hESCs [98].

Importantly, a mechanism involving the oscillatory activation of Notch (mediated by the inhibition of Notch signaling by its transcriptional target HES1) would contribute to the heterogeneous differentiation responses displayed by ESCs. Specifically, cells expressing low or high levels of Hes1 (i.e., with active or inactive Notch signaling, respectively) differentiate preferentially into neural or mesodermal cells, respectively [99–101].

Altogether, this would indicate that expression of Notch components in ESCs would be associated with primed cells displaying a more prone and heterogeneous potential to differentiation, what would be linked to the oscillatory activity of the Notch-Hes1 axis. In turn, the absence of Notch components would be associated with a more homogeneous cell population of naïve cells expressing higher levels of pluripotency factors and with the potential to give rise to the trophectoderm. Up to date, the function of the Notch pathway in the context of the transition between naive and primed states has not been investigated in the literature. However, a recently deposited patent (WO2014174470), defining conditions (referred to as WIS-NHSM) for naive cell generation [102], suggests that Notch inhibitors could contribute to the consolidation of the naive pluripotency state of ESCs [103]. We are currently undertaking this investigation. Thus, although miR-363 is enriched in primed cells [63, 64], inhibition of the Notch pathway may likely be a general mechanism for the maintenance of pluripotency in both naive and primed ESCs.

## Conclusions

By using automated quantitative fluorescence microscopy (i.e., high-content screening) to evaluate the phenotypic effects of microRNA mimics transfected into pluripotent cell lines and by identifying transcripts commonly targeted by microRNAs inducing similar multiparametric phenotypic profiles (as revealed by hierarchical clustering and in-silico target prediction), we were able to identify signaling pathways and biological processes post-transcriptionally modulated by distinct microRNA groups. Integration of this type of data with similar data obtained from siRNA screenings (using the same HCS assay) could provide a large-scale functional approach to identify and validate microRNA-mediated regulatory mechanisms controlling pluripotency and differentiation.

## Additional files

Additional file 1: Supplemental experimental procedures. A detailed description of materials and methods used. (DOCX 52 kb)

Additional file 2: **Table S1.** Synthetic miR mimic molecules used in this study and their miRBase accessions. **Table S2.** List of genes and corresponding TaqMan probe-IDs qPCR. **Table S3.** List of genes and corresponding SYBR Green primers and PCR conditions used for qPCR. **Table S4.** List of Notch pathway components and the corresponding targeting miRNAs. (DOCX 33 kb)

Additional file 3: **Figure S1.** Plate controls of the miRNA screen in NT2 cells. **Figure S2.** Plate controls of the miRNA screen in H1 hESCs. **Figure S3.** Antibody validation. **Figure S4.** Effects of miRNAs on nuclear OCT4 levels in NT2 cells and H1 hESCs cells. **Figure S5.** Effects of miRNAs on cytoplasmic cyclin B1 levels in NT2 cells and H1 hESCs cells. (DOCX 3295 kb)

Additional file 4: DAVID pathways analysis. Excel file with all results from the enrichment pathway analyses carried using DAVID. (XLSX 163 kb)

Additional file 5: Pathways comparisons. Excel file with all comparisons between the pathways identified by DAVID. (XLSX 31 kb)

## Data Availability

Part of the data generated or analyzed during this study are included in this published article [and its supplementary information files]. The remaining datasets used and/or analyzed during the current study are available from the corresponding author on reasonable request.

## Abbreviations

AGO: Argonaute
atRA: All-trans retinoic acid
DAVID: Database for Annotation, Visualization and Integrated Discovery
DL1: Delta-Like 1
EBs: Embryoid bodies
ECCs: Embryonal carcinoma cells
EpiLCs: Epiblast-like cells
ESCC: ES cell–specific cell cycle–regulating
esiRNA: Endoribonuclease-prepared small interfering RNA
HCS: High-content screening
hESCs: Embryonic stem cells
ICM: Inner cell mass
iPSCs: Induced pluripotent stem cells
miR or miRNA: MicroRNA
NICD: Notch intracellular domain
NT2: NTera-2
OSKM: OCT4, SOX2, KLF4, and c-MYC
PMC: Pre-miR negative control
PRC1: Polycomb repressive complexes 1
pre-miRs: Precursor hairpin miRs
pri-miRs: Primary miRs
RISC: RNA-induced silencing complex
siRNA: Small interfering RNA
TFs: Transcription factors
