# Supplementary material for: High-content screen in human pluripotent cells identifies miRNA-regulated pathways controlling pluripotency and differentiation
Source: Stem Cell Res Ther. 2019 Jul 8;10:202. doi: 10.1186/s13287-019-1318-6 (PMC6615276; doi:10.1186/s13287-019-1318-6)
Supplement: Supplementary file 1 — Supplemental experimental procedures. A detailed description of materials and methods used. (DOCX 52 kb) [file 13287_2019_1318_MOESM1_ESM.docx]

## Supplemental Experimental Procedures

### Cell Culture

The NTera-2 cl.D1 cell line (human embryonal carcinoma cells) was obtained from the Cell Bank of Rio de Janeiro (BCRJ) and cultured in DMEM (Dulbecco's Modified Eagle's Medium high glucose, Gibco, #12800-017), supplemented with 10% fetal bovine serum (FBS, GE Life Sciences, # SH30071.03) without antibiotics. The H1 cell line (human embryonic stem cells) was purchased from WiCell and maintained in a feeder-free condition using the GelTrex matrix (Gibco, # A1413302) and the culture medium mTeSR™-1 (STEMCELL Technologies, # 5850). The cells were subcultured with StemPro® Accutase® Dissociation Reagent (ThermoFisher Scientific, #A1110501) and to inhibit spontaneous differentiation as a result from the enzymatic harvesting, a Rock inhibitor reagent (iROCK, 10μM, Hydrochloride Y-27632, Abcam, #ab120129) was used. Both cell lines were maintained in a humidified incubator, with controlled air humidity (85%), temperature (37°C) and CO_2_ concentration (5%). These cell lines were negative for mycoplasma contamination (MycoAlertTM Mycoplasma Detection Kit, Lonza, #LT07) and were investigated by immunofluorescence microscopy for the expression of known pluripotency markers: OCT4 (anti-OCT4, Santa Cruz Biotechnology, #sc-9081), SOX-2 (anti-SOX-2, Santa Cruz , #sc-365823) and TRA-1-81 (anti-TRA-1-81, Santa Cruz Biotechnology, #sc-21706). For some experiments evaluating the effects of miRNAs on Notch activation, the murine OP9-Ctrl and OP9-DL1 cells were used, kindly provided by Dr. Juan-Carlos Zùñiga-Pflücker (University of Toronto, Ontario, Canada). These cells were cultivated in alpha-MEM (ThermoFicher Scientific, #11900-024) with 15% of FBS (GE HealthCare Life Sciences, #SH30071.03) and for subculturing they were harvested with 0.5% trypsin-EDTA (ThermoFisher Scientific, #15400-054).

### miR mimic transfection and culture in 96-well plates

Lipofectamine 2000 (0,05µL/100µL; ThermoFisher Scientific, #11668019) and DharmaFECT-4 (0,1µL/100µL; Dharmacon, #T-2004-03) were used in NT2 and H1 cells, respectively, to transfect 50nM of synthetic miR mimics Pre-miR™ miRNA Precursors (Thermo Fisher Scientific, Table S1), as well as, negative (Pre-miR™ miRNA Precursor Negative Control #1, ThermoFisher, #AM17110) and transfection (siUBC siGenome SMART pool, Dharmacon, #M-019408-01) controls, according to the manufacturer's instructions. For the HCS assay, NT2 (4x10^3^ cells/well) or H1 (6,5x10^3^ cells/well) cells were reversely transfected (in triplicates) in 96-well plates (Sigma-Aldrich, #3603) with 31 miR mimic molecules (individually), selected based on their differential expression between pluripotent cells and their differentiating counterparts (Marson*, et al* 2008, Stadler*, et al* 2010). It is important to notice that prior to transfection, H1 cells were treated for 2h with iROCK and the cells suspension was also prepared in mTeSR-1 with iROCK. After transfection, the cells were incubated for 4 or 3 days (for NT2 and H1, respectively). No media change was carried with NT2 cells. For H1 ESCs, after 24 hours, 100µL/well of fresh mTeSR-1 was added to the plates and after 48 hours the complete volume (200 µL) was replaced by 100µL/well of mTeSR-1.

### Immunofluorescence and image acquisition

After culture, cells were washed twice with PBS (100µL/well) and fixed/permeabilized using 2% formaldehyde in ice-cold methanol solution (50µL/well), followed by a quenching step with glycine (0,1M; 50µL/well) and blocking with 1% of FBS in PBS (50µL/well). Immunofluorescence staining was carried with an overnight incubation with a solution (50μL/well) containing both anti-OCT4 (1:200, Santa Cruz Biotechnology, #sc-5279) and anti-Cyclin B1 (1:200, Santa Cruz Biotechnology, #sc-595). After incubation, the plates were washed three times with PBS and then incubated with a solution (50μL/well) containing the secondary detection antibodies anti-mouse IgG (1:300, conjugated with DyLight488, Thermo Scientific, #35503) and anti-rabbit (1:300, conjugated with DyLight594, #35561), as well as the nuclear Hoechst 33342 (1µM, Thermo Fisher, #H1399) and cytoplasmic CellMask^®^Blue (0,1:1000, Life Technologies, #H32720) fluorescent stains, for 45 minutes at room temperature, protected from the light. Next, the wells were washed three times with PBS and maintained with 100μL of PBS. Images were acquired with an automated ImageXpress^micro^ XLS High Content Screening system (Molecular Devices), using a 10X objective and the excitation/emission filter cubes for DAPI (Hoechst/CellMask Blue), FITC (DyLight488) and Texas Red (DyLight594). The central nine fields were imaged in each well, totaling 27 images per well (9 for each detection cube), 1620 images per plate (60 wells/plate), 3240 images per assay (2 plates) or 28.9GB of data per experiment with each cell line.

### Image and data analysis

Image and data analysis was carried using the open access softwares CellProfiler (cellprofiler.org) (Carpenter*, et al* 2006, Kamentsky*, et al* 2011) and KNIME Analytics Platform (knime.com) (Stoter*, et al* 2013). Staining of the cells with DNA-intercalating fluorescent stain Hoechst 33342 and HCS CellMask Blue allowed the segmentation of the nuclei and cytoplasm to be carried on images acquired in a single channel, using a three classes Otsu algorithm, based in differing fluorescence intensity levels between the nucleus (high), the cytoplasm (intermediary) and the background (low). Once segmented, several morphometrical features, as well as intensity-related measurements (from OCT4 and Cyclin B1 staining), were obtained from the nuclei and cytoplasm of each cell. For all fluorescence intensity measurements, in order to reduce the effects of outlier pixels, affecting the average obtained for each cell, we used only median values (e.g. median nuclear Oct4 intensity). For each parameter quantified, CellProfiler provides a single value corresponding to the median of all measurements obtained from the cell population in a given site (set of images from distinct channels); thus originating, for each miR treatment, 27 values derived from all 9 sites acquired from the 3 replica wells. The data generated by CellProfiler was next exported and processed using KNIME. To this end, we used basic KNIME processing nodes, as well as nodes specifically suited for the analysis of High Content Screening data (“HCS Tools”, community nodes), developed by members of the Technology Development Studio (TDS) of the Max Planck Institute of Cell Biology and Genetics (MPI-CBG, Dresden, Germany). Initially, a quality evaluation was carried using the “Plate Heatmap Viewer” node to check the behavior of the controls in both plates derived from both screenings with NT2 and H1 cell lines. Transfection efficiency was evaluated by comparing the total number of cells (nuclei) in wells with cells transfected with the lethal siRNA-UBC control, to those transfected with miR-Ctr. To further evaluate if both plates of each screening were comparable; for each plate we evaluated the reduction of nuclear OCT4 staining (as compared to cells transfected with miR-Ctr) in the wells with cells transfected with esiRNA against OCT4 (esiOCT4, #EHU223451, Sigma-Aldrich) or treated with 10uM atRA (#R2625, Sigma-Aldrich). In order to minimize the effects of experimental plate-to-plate variation, allowing a direct comparison of all treatment conditions in both plates of each screening, we carried a normalization using reference control wells present in both plates (wells with cells transfected with the control miR-Ctr). To this end, the percentage increase or decrease of a given quantified phenotypic parameter value, relative to the values obtained for the control wells, was calculated as a Percentage of Control (POC) value. Graphs representing a single parameter were plotted in Graphpad Prism, using the POC values derived from all 27 sites acquired from the 3 replica wells, for each miR treatment. In order to obtain a multiparametric phenotypic profile representing the effect of each miRNA in the whole population, a single value was derived from all 27 values for each parameter quantified. To accomplish this, the median from all nine sites in a given well was obtained and next, the median of all three replica wells was used. This allowed us to obtain more robust representative measurements, less prompt to outliers derived from artifacts in a given site or a given well. Finally, we carried a manual feature selection, in order to obtain a less redundant and more representative (as well as more naturally interpretable) set of biologically relevant phenotypic parameters, which together formed the multiparametric profile.

### Phenotypic clustering of miRs, identification of shared predicted targets and pathway analysis

The multiparametric phenotypic profiles obtained for all miR treatments was then submitted to hierarchical clustering using the software Cluster 3.0, using centered correlation metrics and average linkage (de Hoon*, et al* 2004), and heatmaps and cluster were generated and visualized using Java Treeview (Saldanha 2004). Next, given that miRs can act together in a given biological process, by commonly targeting specific components of a given pathway or, alternatively, by targeting distinct components of the same pathway (Hausser and Zavolan 2014, Shalgi*, et al* 2009); we used all predicted mRNA targets of each miRNA, downloaded from TargetScan Human 7.1 (Agarwal*, et al* 2015), to identify and select transcripts commonly targeted by miRs belonging to the same cluster (i.e. inducing a similar phenotypic effect, as evidenced by clustered multiparametric profiles). Finally, we used the Database for Annotation, Visualization and Integrated Discovery (DAVID V6.8, david.ncifcrf.gov) (Dennis*, et al* 2003, Huang da*, et al* 2009), to identify signaling pathways and biological processes showing enrichment for shared targets from miRs of a given phenotypic cluster and, thus, potentially regulated at the post-transcriptional level by these miRs. Given the limitation of the DAVID tool to deal with larger gene lists, we empirically defined the minimum number of miRNAs targeting a given transcript mRNA in order to select it, thus limiting the number of selected shared target transcripts to a maximum of 3000. Specifically, for clusters B.1 and B.2a in NT2, and A and B.1 in H1 (see Figure 3 and 4 in results), the minimum number of miRs targeting each transcript was 3; for clusters C.2b and C.2c in H1, 4 miRs per transcript; for A.2 in NT2 and C.1 in H1, 5 miRNAs per transcript; and for cluster B.2b in NT2, 6 miRNAs per transcript. Worth of notice, few miRs formed an outer branch, equidistant to other two clusters. For the identification of shared targets, these miRs were assigned to the cluster with a more similar phenotype, regarding the effect on OCT4 expression. Thus, miR-302a-5p was included in cluster B.2 in the NT2 screen; while, for the H1 screen, miR-363-3p was assigned to cluster B.1 and miR-23a-3p was assigned to cluster C.1. For the clusters formed exclusively by miR-29a-3p and miR-29a-3p in NT2 (A.3) and H1 (B.2), in order to restrict the total number of shared targets, we eliminated the targets common to the remaining miRs in the pro-differentiation cluster. Once the pathways to each of the phenotypical clusters were identified by DAVID, we used Venny 2.1 (http://bioinfogp.cnb.csic.es/tools/venny/) to compare them and identify pathways exclusive of the pro-pluripotency or pro-differentiation groups of miRNAs, as well as those pathways commonly identified among them.

### Validation of miR targets by qPCR

To validate the regulatory effect of miRNAs on some identified target, NT2 cells (4,5x10^4^ cell/well) were reversely transfected with selected miR mimics in 12-well plates (Greiner Bio-one, #665180) and cultured for 2 days prior to RNA extraction. Total RNA from all samples was prepared using TRIzol LS Reagent (Thermo Fisher Scientific), as described by the manufacturer, and quantified on a Nanovue Plus Spectrophotometer (GE). Total RNA (1µg) was reverse transcribed into cDNA, using the High Capacity cDNA Reverse Transcription Kit (Applied Biosystems, #4368813). The qPCR was performed using either TaqMan Universal PCR Master Mix (Thermo Fisher Scientific, #4304437) or Power SYBR Green PCR Master Mix (ThermoFisher Scientific, #4367659). Relative gene expression was obtained using the 2^-DDCT method (Livak and Schmittgen 2001), by normalizing CT values by *GAPDH* expression (DCT) and then, by using the average DCT value obtained for the control group (miR-Ctr) as a reference value. TaqMan probes and SYBR Green PCR primers used are listed in supplementary tables S2 and S3 (see Additional file 1).

### Regulation of Notch pathway by miRNAs in pluripotent stem cells

To further dissect the potential repression of Notch signaling by miRs promoting pluripotency features, we obtained a list of genes related to the Notch pathway (including components and targets) and identified all miRs targeting them. As a result, we identified four miRNAs with more than ten targets (miR-302c-3p, miR-101-3p, miR-363-3p and miR-92a-3p) and two miRNAs with less than five targets (miR-222-3p and miR-371-3p). NT2 cells (4x10^3^) were transfected in 96-well plates with the mimics for these miRNAs and controls. These cells were incubated for 3 days and then co-cultured for 24h with OP9-ctrl or OP9-DL1 cells (5x10^3^ cell/well) to promote differentiation induced by Notch activation. Next, cells were fixed/permeabilized, stained for OCT4 and imaged as described before. The intensity of OCT4 staining was measured using CellProfiler and the results were compared to the cells transfected with pre-miR negative control and co-cultured with OP9-Ctr.

### Antibody Validation

To validate the quantitative results obtained by the antibodies used in our HCS assay, we compared them to those obtained with distinct antibodies using quantitative microscopy. Western blotting was used to further validate the specificity of selected antibodies and, also, to compare how the quantitative results from HCS relate to those obtained by densitometric analysis of western blot protein bands. For this, NTera-2 cells were cultured as described (DMEM, 10% FBS) and then seeded in 96-well plates for quantitative microscopy (2.5x10^3^ cells/well) or in 15cm culture plates (4.53x10^5^ cells/plate) for protein extraction and western blotting. Twenty four hours after seeding, cells were treated (or not) with atRA (10uM) and cultured for 72 additional hours. Plates were then stained with the following antibodies: polyclonal rabbit anti-OCT4 IgG (sc-9081, Santa Cruz Biotechnology, Santa Cruz, CA; 1:250 dilution), monoclonal mouse anti-OCT4 IgG (sc-5279; 1:150 dilution), monoclonal mouse anti-OCT4 PE-conjugated IgG (BD560186, BD Life Sciences, Franklin Lakes, NJ; 1:100 dilution), polyclonal rabbit anti-cyclin B1 IgG (sc-595; 1:200 dilution) and monoclonal mouse anti-cyclin B1 IgG (sc-245; 1:200 dilution). Following staining with appropriate secondary fluorochrome-conjugated antibodies (except for the PE-conjugated anti-OCT4 antibody), images were acquired using the ImageXpress^micro^ XLS HCS system. The median cell integrated intensity of OCT4 and cyclin B1 staining was measured using CellProfiler and the results were calculated and plotted as percentage of controls (POC), using the median value of the controls as a reference. Of notice, integrated intensities of whole cells were used to allow a more direct comparison between these results and those obtained by western blotting, as the latter technique reflects the total amounts of a given protein in the total cell extract (cytoplasm and nuclei).

For western blotting, only the rabbit anti-OCT4 and anti-cyclin B1 antibodies (sc-9081 and sc-595, respectively) were used. For protein extraction, cells were washed with PBS and disrupted in lyses buffer (20 mM Tris-HCl (pH 7.5), 150 mM NaCl, 1 mM Na2EDTA, 1 mM EGTA, 1% Triton X-100, 2.5 mM sodium pyrophosphate, 1 mM β-glycerophosphate, 1 mM Na3VO4 and1 µg/ml leupeptin). After three sonication cycles (5min at 45W) in a sonicator bath (Unique, São Paulo, Brazil), the samples were centrifuged at 20,000 xg for 30 min at 4°C and the concentration of the protein supernatant was determined by the Bradford method (Bio-Rad, Hercules, CA). Proteins were submitted to SDS–PAGE and electrotransferred to PVDF membranes (GE Lifesciences, Pittsburgh, PA, USA). Membranes were blocked with 5% non-fat dry milk in 0.1% Tween-TBS and incubated with the primary antibodies (anti-cyclin B1 and anti-OCT4) and rabbit anti-GAPDH (#2118, Cell Signaling, Beverly, MA). After 1 hour of incubation with horseradish peroxidase-conjugated goat anti-rabbit IgG secondary antibody (#7074, Cell Signaling). The antibody-protein complex was detected using ECL Western Blotting Detection Reagents (GE Lifesciences) using a CCD-Camera (Image QuantLAS 4000 mini, Uppsala, Sweden). Densitometric analysis was performed using the ImageJ software (Schneider*, et al* 2012). The amount of protein loaded in each lane was normalized by the total intensity of the constitutive protein GAPDH. The relative reduction in the protein levels of OCT4 and cyclin B1, in the atRA-treated samples, was calculated using the control samples as references.

## Supplemental References

Agarwal, V., Bell, G.W., Nam, J.W. & Bartel, D.P. (2015) Predicting effective microRNA target sites in mammalian mRNAs. *Elife,* **4**.

Carpenter, A.E., Jones, T.R., Lamprecht, M.R., Clarke, C., Kang, I.H., Friman, O., Guertin, D.A., Chang, J.H., Lindquist, R.A., Moffat, J., Golland, P. & Sabatini, D.M. (2006) CellProfiler: image analysis software for identifying and quantifying cell phenotypes. *Genome Biol,* **7,** R100.

de Hoon, M.J., Imoto, S., Nolan, J. & Miyano, S. (2004) Open source clustering software. *Bioinformatics,* **20,** 1453-1454.

Dennis, G., Jr., Sherman, B.T., Hosack, D.A., Yang, J., Gao, W., Lane, H.C. & Lempicki, R.A. (2003) DAVID: Database for Annotation, Visualization, and Integrated Discovery. *Genome Biol,* **4,** P3.

Hausser, J. & Zavolan, M. (2014) Identification and consequences of miRNA-target interactions--beyond repression of gene expression. *Nat Rev Genet,* **15,** 599-612.

Huang da, W., Sherman, B.T. & Lempicki, R.A. (2009) Systematic and integrative analysis of large gene lists using DAVID bioinformatics resources. *Nat Protoc,* **4,** 44-57.

Kamentsky, L., Jones, T.R., Fraser, A., Bray, M.A., Logan, D.J., Madden, K.L., Ljosa, V., Rueden, C., Eliceiri, K.W. & Carpenter, A.E. (2011) Improved structure, function and compatibility for CellProfiler: modular high-throughput image analysis software. *Bioinformatics,* **27,** 1179-1180.

Livak, K.J. & Schmittgen, T.D. (2001) Analysis of relative gene expression data using real-time quantitative PCR and the 2(-Delta Delta C(T)) Method. *Methods,* **25,** 402-408.

Marson, A., Levine, S.S., Cole, M.F., Frampton, G.M., Brambrink, T., Johnstone, S., Guenther, M.G., Johnston, W.K., Wernig, M., Newman, J., Calabrese, J.M., Dennis, L.M., Volkert, T.L., Gupta, S., Love, J., Hannett, N., Sharp, P.A., Bartel, D.P., Jaenisch, R. & Young, R.A. (2008) Connecting microRNA genes to the core transcriptional regulatory circuitry of embryonic stem cells. *Cell,* **134,** 521-533.

Saldanha, A.J. (2004) Java Treeview--extensible visualization of microarray data. *Bioinformatics,* **20,** 3246-3248.

Schneider, C.A., Rasband, W.S. & Eliceiri, K.W. (2012) NIH Image to ImageJ: 25 years of image analysis. *Nat Methods,* **9,** 671-675.

Shalgi, R., Brosh, R., Oren, M., Pilpel, Y. & Rotter, V. (2009) Coupling transcriptional and post-transcriptional miRNA regulation in the control of cell fate. *Aging (Albany NY),* **1,** 762-770.

Stadler, B., Ivanovska, I., Mehta, K., Song, S., Nelson, A., Tan, Y., Mathieu, J., Darby, C., Blau, C.A., Ware, C., Peters, G., Miller, D.G., Shen, L., Cleary, M.A. & Ruohola-Baker, H. (2010) Characterization of microRNAs involved in embryonic stem cell states. *Stem Cells Dev,* **19,** 935-950.

Stoter, M., Niederlein, A., Barsacchi, R., Meyenhofer, F., Brandl, H. & Bickle, M. (2013) CellProfiler and KNIME: open source tools for high content screening. *Methods Mol Biol,* **986,** 105-122.
