# Supplementary material for: High-content screen in human pluripotent cells identifies miRNA-regulated pathways controlling pluripotency and differentiation
Source: Stem Cell Res Ther. 2019 Jul 8;10:202. doi: 10.1186/s13287-019-1318-6 (PMC6615276; doi:10.1186/s13287-019-1318-6)
Supplement: Supplementary file 2 — Table S1. Synthetic miR mimic molecules used in this study and their miRBase accessions. Table S2. List of genes and corresponding TaqMan probe-IDs qPCR. Table S3. List of genes and corresponding SYBR Green primers and PCR conditions used for qPCR. Table S4. List of Notch pathway components and the corresponding targeting miRNAs. (DOCX 33 kb) [file 13287_2019_1318_MOESM2_ESM.docx]

**Additional file 2**

**Table S1.** Synthetic miR mimic molecules used in this study and their miRBase accessions.

| Name_Ambion | Acc_Mat. | ID_Ambion | Name_miRBase | Mature Sequence | Family | Seed | Conservation |
| --- | --- | --- | --- | --- | --- | --- | --- |
| hsa-miR-17* | MIMAT0000071 | PM12246 | hsa-miR-17-3p | ACUGCAGUGAAGGCACUUGUAG | miR-17-3p | CUGCAGU | 0 |
| hsa-miR-18a | MIMAT0000072 | PM12973 | hsa-miR-18a-5p | UAAGGUGCAUCUAGUGCAGAUAG | miR-18-5p/4735-3p | AAGGUGC | 2 |
| hsa-miR-18b | MIMAT0001412 | PM10466 | hsa-miR-18b-5p | UAAGGUGCAUCUAGUGCAGUUAG | miR-18-5p/4735-3p | AAGGUGC | 2 |
| hsa-miR-19a | MIMAT0000073 | PM10649 | hsa-miR-19a-3p | UGUGCAAAUCUAUGCAAAACUGA | miR-19-3p | GUGCAAA | 2 |
| hsa-miR-19b | MIMAT0000074 | PM10629 | hsa-miR-19b-3p | UGUGCAAAUCCAUGCAAAACUGA | miR-19-3p | GUGCAAA | 2 |
| hsa-miR-20a | MIMAT0000075 | PM10057 | hsa-miR-20a-5p | UAAAGUGCUUAUAGUGCAGGUAG | miR-17-5p/20-5p/93-5p/106-5p/519-3p/526-3p | AAAGUGC | 2 |
| hsa-miR-20b | MIMAT0001413 | PM10975 | hsa-miR-20b-5p | CAAAGUGCUCAUAGUGCAGGUAG | miR-17-5p/20-5p/93-5p/106-5p/519-3p/526-3p | AAAGUGC | 2 |
| hsa-miR-21 | MIMAT0000076 | PM10206 | hsa-miR-21-5p | UAGCUUAUCAGACUGAUGUUGA | miR-21-5p/590-5p | AGCUUAU | 2 |
| hsa-miR-22 | MIMAT0000077 | PM10203 | hsa-miR-22-3p | AAGCUGCCAGUUGAAGAACUGU | miR-22-3p | AGCUGCC | 2 |
| hsa-miR-23a | MIMAT0000078 | PM10644 | hsa-miR-23a-3p | AUCACAUUGCCAGGGAUUUCC | miR-23-3p/130a-5p | UCACAUU | 2 |
| hsa-miR-24 | MIMAT0000080 | PM10737 | hsa-miR-24-3p | UGGCUCAGUUCAGCAGGAACAG | miR-24-3p | GGCUCAG | 2 |
| hsa-miR-27a | MIMAT0000084 | PM10939 | hsa-miR-27a-3p | UUCACAGUGGCUAAGUUCCGC | miR-27-3p | UCACAGU | 2 |
| hsa-miR-29a | MIMAT0000086 | PM12499 | hsa-miR-29a-3p | UAGCACCAUCUGAAAUCGGUUA | miR-29-3p | AGCACCA | 2 |
| hsa-miR-29b | MIMAT0000100 | PM10103 | hsa-miR-29b-3p | UAGCACCAUUUGAAAUCAGUGUU | miR-29-3p | AGCACCA | 2 |
| hsa-miR-30a | MIMAT0000087 | PM11062 | hsa-miR-30a-5p | UGUAAACAUCCUCGACUGGAAG | miR-30-5p | GUAAACA | 2 |
| hsa-miR-92a | MIMAT0000092 | PM10916 | hsa-miR-92a-3p | UAUUGCACUUGUCCCGGCCUGU | miR-25-3p/32-5p/92-3p/363-3p/367-3p | AUUGCAC | 2 |
| hsa-miR-101 | MIMAT0000099 | PM11414 | hsa-miR-101-3p | UACAGUACUGUGAUAACUGAA | miR-101-3p.1 | ACAGUAC | 2 |
| hsa-miR-106a | MIMAT0000103 | PM12567 | hsa-miR-106a-5p | AAAAGUGCUUACAGUGCAGGUAG | miR-17-5p/20-5p/93-5p/106-5p/519-3p/526-3p | AAAGUGC | 2 |
| hsa-miR-145 | MIMAT0000437 | PM11480 | hsa-miR-145-5p | GUCCAGUUUUCCCAGGAAUCCCU | miR-145-5p/5195-3p | UCCAGUU | 2 |
| hsa-miR-181d | MIMAT0002821 | PM12522 | hsa-miR-181d-5p | AACAUUCAUUGUUGUCGGUGGGU | miR-181-5p/4262 | ACAUUCA | 2 |
| hsa-miR-222 | MIMAT0000279 | PM11376 | hsa-miR-222-3p | AGCUACAUCUGGCUACUGGGU | miR-221-3p/222-3p | GCUACAU | 2 |
| hsa-miR-302a | MIMAT0000684 | PM10936 | hsa-miR-302a-3p | UAAGUGCUUCCAUGUUUUGGUGA | miR-302-3p/372-3p/373-3p/520-3p | AAGUGCU | 2 |
| hsa-miR-302a* | MIMAT0000683 | PM12557 | hsa-miR-302a-5p | ACUUAAACGUGGAUGUACUUGCU | miR-302a-5p | CUUAAAC | 0 |
| hsa-miR-302b | MIMAT0000715 | PM10081 | hsa-miR-302b-3p | UAAGUGCUUCCAUGUUUUAGUAG | miR-302-3p/372-3p/373-3p/520-3p | AAGUGCU | 2 |
| hsa-miR-302b* | MIMAT0000714 | PM12916 | hsa-miR-302b-5p | ACUUUAACAUGGAAGUGCUUUC | miR-302bd-5p | CUUUAAC | 0 |
| hsa-miR-302c | MIMAT0000717 | PM10571 | hsa-miR-302c-3p | UAAGUGCUUCCAUGUUUCAGUGG | miR-302-3p/372-3p/373-3p/520-3p | AAGUGCU | 2 |
| hsa-miR-302d | MIMAT0000718 | PM10927 | hsa-miR-302d-3p | UAAGUGCUUCCAUGUUUGAGUGU | miR-302-3p/372-3p/373-3p/520-3p | AAGUGCU | 2 |
| hsa-miR-363 | MIMAT0000707 | PM10149 | hsa-miR-363-3p | AAUUGCACGGUAUCCAUCUGUA | miR-25-3p/32-5p/92-3p/363-3p/367-3p | AUUGCAC | 2 |
| hsa-miR-371-3p | MIMAT0000723 | PM12262 | hsa-miR-371a-3p | AAGUGCCGCCAUCUUUUGAGUGU | miR-371a-3p | AGUGCCG | 0 |
| hsa-miR-372 | MIMAT0000724 | PM10165 | hsa-miR-372-3p | AAAGUGCUGCGACAUUUGAGCGU | miR-302-3p/372-3p/373-3p/520-3p | AAGUGCU | 2 |
| hsa-miR-373 | MIMAT0000726 | PM11024 | hsa-miR-373-3p | GAAGUGCUUCGAUUUUGGGGUGU | miR-302-3p/372-3p/373-3p/520-3p | AAGUGCU | 2 |

Acc_Mat. = mature miRNA miRBase accession number; Name_miRBase = miRBase nomenclature. Conservation column indicates that a miRNA family is highly conserved (2), conserved (1), or poorly conserved (0). Families, seed and conservation data were derived from TargetScan 7.

**Table S2.** List of genes and corresponding TaqMan probe-IDs used for qPCR.

| Gene | TaqMan Probe | AT |
| --- | --- | --- |
| APC | Hs00181051_m1 | 60°C |
| BMPR2 | Hs00176148_m1 | 60°C |
| CDKN2B | Hs00365249_m1 | 60°C |
| FGFR1 | Hs00241111_m1 | 60°C |
| GSK3B | Hs00275656_m1 | 60°C |
| IGF1R | Hs00609566_m1 | 60°C |
| MAPK1 | Hs01046830_m1 | 60°C |
| PTEN | Hs00829813_s1 | 60°C |
| RELA | Hs00153294_m1 | 60°C |
| STAT3 | HS00374280-m1 | 60°C |
| TGFB3 | Hs01086000_m1 | 60°C |
| TGFBR2 | HS00234253_m1 | 60°C |

AT = annealing temperature

**Table S3.** List of genes, primers and qPCR conditions.

| Gene | Primer Sequence (5’–3’) | Concentration | AT |
| --- | --- | --- | --- |
| APC | F: CAGGAAGTATTGAAGATGAAGCTATG  R: CCATAAGAACGGAGGGACATT | 400nM | 62°C |
| CDK6 | F: GTGCCCACTGAAACCATAAAG  R: GTCAGCGAGTTTTATTTGTCCG | 400nM | 62°C |
| GAPDH | F: GAAGGTGAAGGTCGGAGTC  R: GAAGATGGTGATGGGATTTC | 200nM | 60°C |
| GSK3β | F: GGTCTATCTTAATCTGGTGCTGG  R: TGGATATAGGCTAAACTTCGGAAC | 400nM | 62°C |
| IL2RA | F: TCATCTCATTCCAACTTCCCAG  R: TGAGAAAGGAACCACGCAG | 100nM | 60°C |
| IL6ST | F: GCAACATTCTTACATTCGGACAG  R: TCCCACTCACACCTCATTTTC | 150nM | 62,5°C |
| KLF4 | F: GATGAACTGACCAGGCACTA  R: GTGGGTCATATCCACTGTCT | 400nM | 60°C |
| LIF | F: AGTATAAGCAGATCATCGCCG  R: ATTTGGGTTTAGCGATGCC | 400nM | 60°C |
| RELA | F: TGACAAGGTGCAGAAAGAGG  R: CACATCAGCTTGCGAAAAGG | 300nM | 62°C |
| SMAD2 | F: GGTGTGAGAAAGCAGTGAAAAG  R: ACTCAGTCCCCAAATTTCAGAG | 400nM | 62°C |
| TCF3 | F: TTCTCGTCCAGCCCTTCTA  R: GGTGGTCTTCTATCTTACTCTGC | 400nM | 62°C |

AT = annealing temperature.

**Table S4.** List of Notch pathway components and the corresponding targeting miRNAs.

| Gene Name | Predicted targeting miRNAs |
| --- | --- |
| ADAM17 | miR-101-3p, miR-145-5p, miR-181d-5p, miR-222-3p, miR-23a-3p, miR-24-3p, miR-302a-3p, miR-302b-3p, miR-302c-3p, miR-302d-3p, miR-372-3p, miR-373-3p |
| APH1A | miR-145-5p, miR-24-3p, miR-302a-3p, miR-302b-3p, miR-302c-3p, miR-302d-3p, miR-372-3p, miR-373-3p |
| APH1B | miR-101-3p, miR-145-5p, miR-17-3p, miR-181d-5p, miR-18a-5p, miR-18b-5p, miR-21-5p, miR-23a-3p, miR-24-3p, miR-302c-3p, miR-363-3p, miR-92a-3p |
| CIR1 | miR-101-3p, miR-21-5p, miR-302a-5p |
| CREBBP | miR-106a-5p, miR-20a-5p, miR-20b-5p, miR-23a-3p, miR-24-3p, miR-302a-3p, miR-302a-5p, miR-302b-3p, miR-302b-5p, miR-302c-3p, miR-302d-3p, miR-372-3p, miR-373-3p |
| CTBP1 | miR-101-3p, miR-17-3p, miR-302b-5p |
| CTBP2 | miR-101-3p, miR-181d-5p, miR-18a-5p, miR-18b-5p, miR-23a-3p, miR-302a-5p, miR-302b-5p |
| DLL1 | miR-24-3p, miR-371a-3p |
| DLL4 | miR-101-3p, miR-302a-5p, miR-302c-3p, miR-363-3p, miR-92a-3p |
| DTX1 | miR-145-5p |
| DTX2 | miR-363-3p, miR-92a-3p |
| DTX3 | miR-24-3p |
| DTX3L | miR-101-3p, miR-17-3p, miR-21-5p, miR-222-3p, miR-24-3p, miR-302b-5p |
| DTX4 | miR-145-5p, miR-17-3p, miR-181d-5p, miR-24-3p, miR-302a-3p, miR-302b-3p, miR-302c-3p, miR-302d-3p, miR-363-3p, miR-372-3p, miR-373-3p, miR-92a-3p |
| DVL1 | miR-101-3p, miR-18a-5p, miR-18b-5p |
| DVL2 | miR-222-3p, miR-23a-3p |
| DVL3 | miR-106a-5p, miR-18a-5p, miR-18b-5p, miR-20a-5p, miR-20b-5p, miR-21-5p, miR-24-3p, miR-302a-3p, miR-302b-3p, miR-302c-3p, miR-302d-3p, miR-372-3p, miR-373-3p |
| EP300 | miR-106a-5p, miR-20a-5p, miR-20b-5p |
| HDAC1 | miR-24-3p |
| HDAC2 | miR-145-5p, miR-17-3p, miR-21-5p, miR-23a-3p, miR-24-3p, miR-302a-5p, miR-302b-5p, miR-363-3p, miR-92a-3p |
| JAG1 | miR-17-3p, miR-21-5p, miR-23a-3p, miR-302b-5p |
| JAG2 | miR-145-5p, miR-19a-3p, miR-19b-3p, miR-24-3p, miR-302c-3p |
| KAT2B | miR-106a-5p, miR-181d-5p, miR-19a-3p, miR-19b-3p, miR-20a-5p, miR-20b-5p, miR-23a-3p, miR-302a-3p, miR-302b-3p, miR-302c-3p, miR-302d-3p, miR-363-3p, miR-372-3p, miR-373-3p, miR-92a-3p |
| LFNG | miR-24-3p |
| MAML1 | miR-145-5p, miR-17-3p, miR-19a-3p, miR-19b-3p, miR-222-3p, miR-23a-3p, miR-24-3p, miR-302a-3p, miR-302b-3p, miR-302b-5p, miR-302c-3p, miR-302d-3p, miR-372-3p, miR-373-3p |
| MAML2 | miR-101-3p, miR-17-3p, miR-23a-3p, miR-302a-3p, miR-302a-5p, miR-302b-3p, miR-302b-5p, miR-302c-3p, miR-302d-3p, miR-363-3p, miR-372-3p, miR-373-3p, miR-92a-3p |
| MAML3 | miR-101-3p, miR-145-5p, miR-17-3p, miR-18a-5p, miR-18b-5p, miR-19a-3p, miR-19b-3p, miR-24-3p, miR-302a-5p, miR-302b-5p, miR-363-3p, miR-92a-3p |
| MFNG | miR-302a-5p |
| NCSTN | miR-145-5p, miR-24-3p |
| NOTCH1 | miR-101-3p, miR-363-3p, miR-92a-3p |
| NOTCH2 | miR-101-3p, miR-181d-5p, miR-18a-5p, miR-18b-5p, miR-19a-3p, miR-19b-3p, miR-23a-3p, miR-302a-3p, miR-302a-5p, miR-302b-3p, miR-302c-3p, miR-302d-3p, miR-363-3p, miR-372-3p, miR-373-3p, miR-92a-3p |
| NOTCH3 | miR-24-3p |
| NOTCH4 | miR-181d-5p, miR-302a-5p |
| NUMB | miR-101-3p, miR-17-3p |
| NUMBL | miR-106a-5p, miR-20a-5p, miR-20b-5p, miR-302a-3p, miR-302b-3p, miR-302c-3p, miR-302d-3p, miR-372-3p, miR-373-3p |
| PSEN1 | miR-101-3p, miR-106a-5p, miR-145-5p, miR-17-3p, miR-181d-5p, miR-18a-5p, miR-18b-5p, miR-20a-5p, miR-20b-5p, miR-222-3p, miR-24-3p, miR-302a-3p, miR-302b-3p, miR-302b-5p, miR-302c-3p, miR-302d-3p, miR-363-3p, miR-372-3p, miR-373-3p, miR-92a-3p |
| PSEN2 | miR-302a-5p |
| RBPJ | miR-101-3p, miR-181d-5p, miR-18a-5p, miR-18b-5p, miR-21-5p, miR-302a-5p, miR-302b-5p, miR-302c-3p, miR-363-3p, miR-92a-3p |
| RBPJL | miR-17-3p |
| RFNG | miR-17-3p, miR-302a-3p, miR-302a-5p, miR-302b-3p, miR-302c-3p, miR-302d-3p, miR-372-3p, miR-373-3p |
| SNW1 | miR-145-5p, miR-302b-5p, miR-302c-3p |
