# Supplementary material for: High-content screen in human pluripotent cells identifies miRNA-regulated pathways controlling pluripotency and differentiation
Source: Stem Cell Res Ther. 2019 Jul 8;10:202. doi: 10.1186/s13287-019-1318-6 (PMC6615276; doi:10.1186/s13287-019-1318-6)
Supplement: Supplementary file 3 — Figure S1. Plate controls of the miRNA screen in NT2 cells. Figure S2. Plate controls of the miRNA screen in H1 hESCs. Figure S3. Antibody validation. Figure S4. Effects of miRNAs on nuclear OCT4 levels in NT2 cells and H1 hESCs cells. Figure S5. Effects of miRNAs on cytoplasmic cyclin B1 levels in NT2 cells and H1 hESCs cells. (DOCX 3295 kb) [file 13287_2019_1318_MOESM3_ESM.docx]

## Additional file 3

**
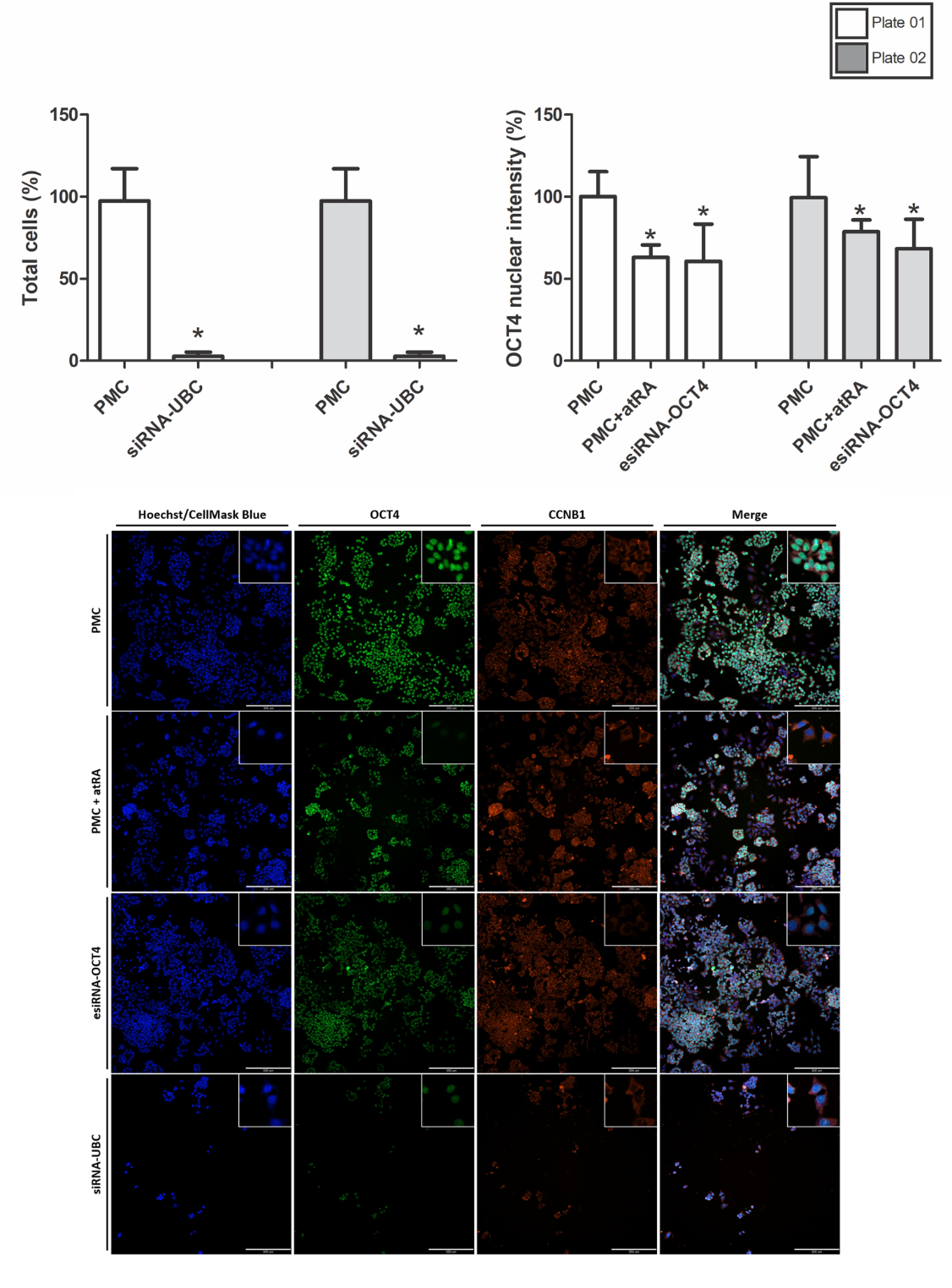
**

**Figure S1. Plate controls of the miRNA screen in NT2 cells.** In each of the two screen plated used, NT2 cells were transfected with negative control miR-Ctr (PMC), a cytotoxic siRNA against UBC (siRNA-UBC), an esiRNA against OCT4 (esiRNA-OCT4), or induced to differentiate by treatment with all-trans retinoic acid (atRA). After quantitative fluorescence microscopy, observed cell counts and OCT4 nuclear median intensity were represented as a percentage relative to the values observed in cells transfected with PMC. Top left Graph: transfection efficiency in both plates, as indicated by the percentage reduction in the number of cells caused by the cytotoxic siRNA-UBC effect. Top right: Effect of Oct4 knockdown (esiRNA-OCT4) or atRA-induced differentiation (atRA) in both screen plates. Bottom panel: representative photomicrographies of the controls.

**
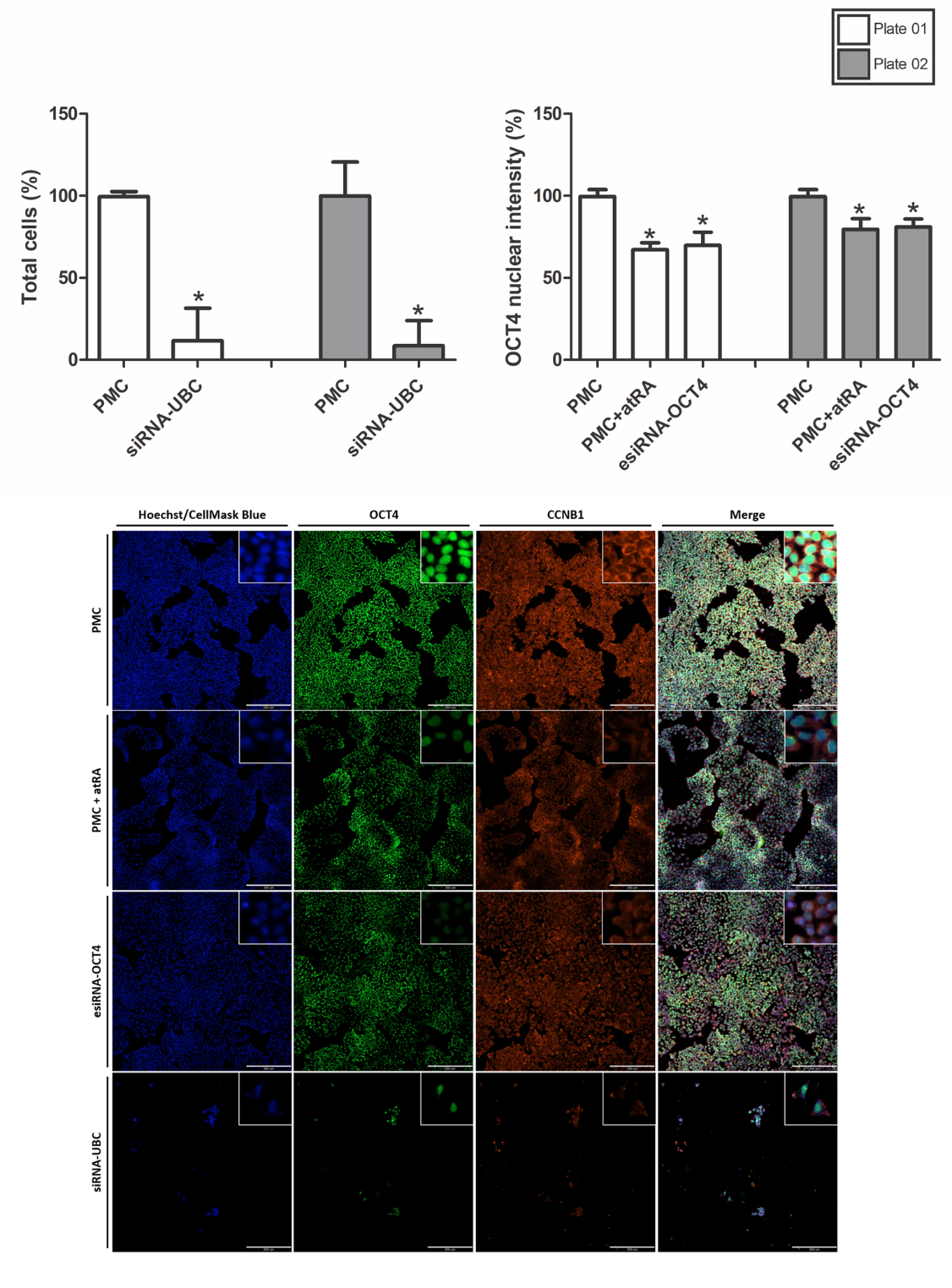
**

**Figure S2.** **Plate controls of the miRNA screen in H1 hESCs.** In each of the two screen plated used, H1 hESCs were transfected with negative control miR-Ctr (PMC), a cytotoxic siRNA against UBC (siRNA-UBC), an esiRNA against OCT4 (esiRNA-OCT4), or induced to differentiate by treatment with all-trans retinoic acid (atRA). After quantitative fluorescence microscopy, observed cell counts and OCT4 nuclear median intensity were represented as a percentage relative to the values observed in cells transfected with PMC. Top left Graph: transfection efficiency in both plates, as indicated by the percentage reduction in the number of cells caused by the cytotoxic siRNA-UBC effect. Top right: Effect of Oct4 knockdown (esiRNA-OCT4) or atRA-induced differentiation (atRA) in both screen plates. Bottom panel: representative photomicrographies of the controls.

**

**

**Figure S3.** **Antibody validation.** NTera-2 cells were seeded in 96-well plates for HCS quantitative microscopy (n=5) or in 15cm culture plates for western blotting (n=2). Twenty four hours after seeding, cells were treated (or not) with atRA and cultured for 72 additional hours. A) Plates were stained with distinct anti-OCT4 and anti-cyclin B1 antibodies and following staining with appropriate secondary fluorochrome-conjugated antibodies (except for the PE-conjugated anti-OCT4 antibody), images were acquired using the ImageXpress^micro^ XLS HCS system and the median integrated intensity of OCT4 (Left) and cyclin B1 (Right) staining in the whole cells were measured using CellProfiler. The results were calculated and plotted as percentage of controls (POC), using the median value of the controls as a reference. GraphPad Prism was used to apply a nonparametric Mann-Whitney test, comparing controls and corresponding treatments. B) Folowing protein extraction and SDS–PAGE, western blot was carried using two of the antibodies evaluated in “A)” (both marked with an asterisk). Densitometric analysis was performed using the ImageJ software. The amount of protein loaded in each lane was normalized by the total intensity of GAPDH. The relative reduction in the protein levels of OCT4 and cyclin B1, in the atRA-treated samples, was calculated using the control samples as references.

**

**

**Figure S4.** **Effects of miRNAs on nuclear OCT4 levels in NT2 cells and H1 hESCs cells.** NT2 cells (A) or H1 hESCs cells (B) were transfected with miR mimics and the negative control miR-Ctr (control) and then submitted to quantitative fluorescence microscopy. OCT4 nuclear median intensity is represented as a percentage relative to the value observed in cells transfected with a control miR. Each dot represents the median nuclear OCT4 value of all the cells in a single image from one of the nine acquired sites in each of the three replica wells in the screening plates (totaling 27 measurements). The miRs are ordered as they appear in Figure 2A.

**

**

**Figure S5.** **Effects of miRNAs on cytoplasmic cyclin B1 levels in NT2 cells and H1 hESCs cells.** NT2 cells (A) or H1 hESCs cells (B) were transfected with miR mimics and the negative control miR-Ctr (control) and then submitted to quantitative fluorescence microscopy. cytoplasmic cyclin B1 median intensity is represented as a percentage relative to the value observed in cells transfected with a control miR. Each dot represents the median cytoplasmic cyclin B1 value of all the cells in a single image from one of the nine acquired sites in each of the three replica wells in the screening plates (totaling 27 measurements). The miRs are ordered as they appear in Figure 2A.
